# Targeting Progression in Pulmonary Fibrosis: An Overview of Underlying Mechanisms, Molecular Biomarkers, and Therapeutic Intervention

**DOI:** 10.3390/life14020229

**Published:** 2024-02-06

**Authors:** Vito D’Agnano, Domenica Francesca Mariniello, Michela Ruotolo, Gianluca Quarcio, Alessandro Moriello, Stefano Conte, Antonio Sorrentino, Stefano Sanduzzi Zamparelli, Andrea Bianco, Fabio Perrotta

**Affiliations:** 1 Department of Translational Medical Sciences, University of Campania L. Vanvitelli, 80131 Naples, Italy; vito.dagnano@studenti.unicampania.it (V.D.); domenicafrancesca.mariniello@studenti.unicampania.it (D.F.M.); michela.ruotolo@studenti.unicampania.it (M.R.); gianluca.quarcio@studenti.unicampania.it (G.Q.); alessandro.moriello@studenti.unicampania.it (A.M.); stefano.conte@unicampania.it (S.C.); antonio.sorrentino@studenti.unicampania.it (A.S.); andrea.bianco@unicampania.it (A.B.); 2 Division of Pneumology, A. Cardarelli Hospital, 80131 Naples, Italy; stefano.sanduzzizamparelli@aocardarelli.it

**Keywords:** progressive pulmonary fibrosis, biomarkers, antifibrotics, nintedanib, pirfenidone

## Abstract

Interstitial lung diseases comprise a heterogenous range of diffuse lung disorders, potentially resulting in pulmonary fibrosis. While idiopathic pulmonary fibrosis has been recognized as the paradigm of a progressive fibrosing interstitial lung disease, other conditions with a progressive fibrosing phenotype characterized by a significant deterioration of the lung function may lead to a burden of significant symptoms, a reduced quality of life, and increased mortality, despite treatment. There is now evidence indicating that some common underlying biological mechanisms can be shared among different chronic fibrosing disorders; therefore, different biomarkers for disease-activity monitoring and prognostic assessment are under evaluation. Thus, understanding the common pathways that induce the progression of pulmonary fibrosis, comprehending the diversity of these diseases, and identifying new molecular markers and potential therapeutic targets remain highly crucial assignments. The purpose of this review is to examine the main pathological mechanisms regulating the progression of fibrosis in interstitial lung diseases and to provide an overview of potential biomarker and therapeutic options for patients with progressive pulmonary fibrosis.

## 1. Introduction 

Interstitial lung diseases (ILDs) encompass a large and heterogeneous group of disorders characterized by inflammation and fibrosis of the lung parenchyma, resulting in a stiffening of the lungs [1,2]. Fibrosing-ILD (F-ILD) is characterized by fibrosis detected on pathology or by the presence of honeycombing and/or traction bronchiectasis on high-resolution computed tomography (HRCT) [3]. Although idiopathic pulmonary fibrosis (IPF) is the archetypal F-ILD with a progressive phenotype, a proportion of patients with other chronic fibrosing ILDs, such as chronic hypersensitivity pneumonitis (HP), connective tissue disease-associated interstitial lung diseases (CTD-ILDs), pulmonary sarcoidosis, and idiopathic non-specific interstitial pneumonia (iNSIP), may experience a similar clinical course [4,5].

While many shared characteristics between IPF’s and other F-ILDs’ progressive phenotype have been recognized, some features in terms clinical, radiological, and histopathological presentations may diverge [6]. From a pathophysiological perspective, in IPF and in other non-IPF ILDs, fibrosis is the final result, following the gradual loss of epithelial barrier integrity. Although the etiology of epithelial injuries generally differs, arising from autoimmune mechanisms, chronic inhaled organic particles, or an unknown injury in IPF patients and a mounting fibrotic response, is likely to be related with repetitive local micro-injuries and the aberrant repair/regeneration of the epithelial barrier [7]. 

This progressive phenotype is characterized by a deterioration of respiratory symptoms, a decline in pulmonary function tests, ongoing radiological progression, a significantly reduced quality of life, and higher mortality rates, despite the underlying treatment of the disorders and the elimination of disease-promoting stimuli [8,9,10]. 

The term ‘progressive’ has been used for a long time in clinical and research scenarios; however, the nomenclature of progression for the fibrotic phenotype has varied based on worsening symptoms, pulmonary function, or imaging. More recently, the Official ATS/ERS/JRS/ALAT Clinical Practice Guideline clarified the definition of progressive pulmonary fibrosis (PPF), identifying PF-ILDs other than IPF with radiological evidence of pulmonary fibrosis that satisfy at least two out of the following three criteria within a 12-month period: worsening respiratory symptoms, an absolute decline in forced vital capacity (FVC) of 5% or more or an absolute decline in diffusion capacity for carbon monoxide (DLCO) of 10% or more within the previous year, and increased fibrosis on chest CTs [4].

Epidemiologic studies on IPF have documented prevalence rates ranging from 1.3 to 42.7/100,000 people and incidence rates ranging from 2.8 to 19/100,000 people–year. Five-year survival rates have been estimated between 20% and 40%, with a median survival time of 2–5 years from diagnosis [6,11,12]. Evidence about the epidemiological data for PPF patients is still limited. Hambly et al. evaluated the incidence of progression in patients with fibrotic ILD during a 24-month follow-up [13]. They found that out of 2.746 patients with fibrotic ILD, 50% met the PF-ILD ATS/ERS/JRS/ALAT criteria. In particular, progression was developed in 59% of patients with IPF, 58% of patients with f-HP, 51% of patient with unclassifiable ILD (U-ILD), and 45% of patients with CTD-ILDs. The estimated prevalence of PPF ranges from 6.9 in European countries to 70.3 in the U.S./100,000 people, while the estimated incidence ranges from 2.1 in European countries to 32.6 in the U.S./100,000 people–years. Moreover, the percentages of patients with PF-ILDs, other than IPF, harboring a progressive fibrosing phenotype ranges from 10.4 to 60.6%, and the median survival is approximately three years, which is similar to IPF patients [14,15,16].

Despite the different etiologies among F-ILDs, there are often similarities in their underlying pathological mechanisms and clinical and radiological features. In the present review, the pathological mechanisms and key players of signaling pathways involved in fibrosis progression as well as molecular markers and promising therapeutic targets to prevent and/or tackle fibrosis progression are discussed.

## 2. Molecular Mechanisms of Pulmonary Fibrosis

**Figure 1:**
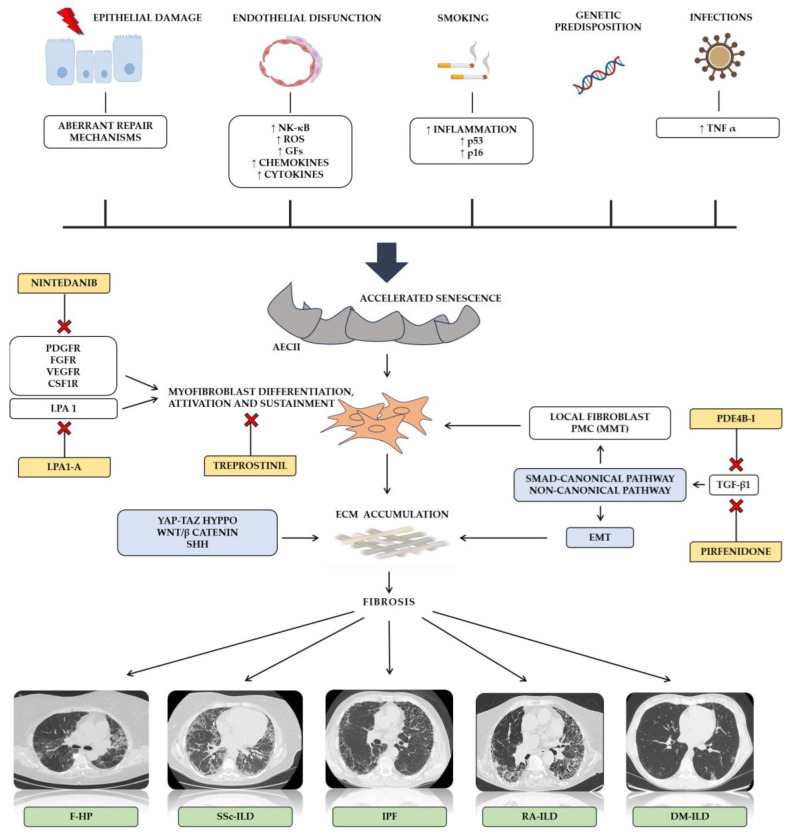
Biological and immunological mechanisms of pulmonary fibrosis. AECII: alveolar type II cells; DM: dermatomyositis; ECM: extracellular matrix; EMT: epithelial–mesenchymal transition; F-HP: fibrotic hypersensitivity pneumonitis; GFs: growth factors; IL: Interleukin; ILD: interstitial lung disease; IPF: idiopathic pulmonary fibrosis; LPA 1: lysophosphatidic acid 1; LOXL: Lysyl oxidase homolog; MMT: mesothelial–mesenchymal transition; NK-κB: nuclear factor kappa-light-chain enhancer of activated B cells; PCMs: pleural mesothelial cells; PDE4B-I: phosphodiesterase 4B inhibitor; PDGF: platelet-derived growth factor; RA: rheumatoid arthritis; ROS: Reactive oxygen species; SSc: systemic sclerosis; SHH: Sonic hedgehog; TNFα: tumor necrosis factor alpha; TAZ: transcriptional coactivator with PDZ-binding motif; YAP: Yes-associated protein.

The molecular distinction between IPF and other PF-ILDs is of profound importance not only because IPF is characterized by a poorer prognosis compared to other PF-ILDs [17]. It also critical for the proper management of patients and for developing new therapeutic strategies [18] (Figure 1). Driven by an increase of inflammation and an activation of p16-pRb and p53 pathways, smoking represent a well-established risk factor for both epithelial damage and AECII-accelerated senescence [19]. Likewise, endothelial dysfunction has been associated with a heterogenous release of pro-inflammatory markers, growth factors, chemokines, and cytokines [20]. In a similar manner, lung infection may contribute to the development of cellular senescence [21]. Regarding IPF, a recurring alveolar epithelium injury with a depletion of alveolar epithelial cells 1 (AECI) seems to be the first step for initiating fibrotic changes. Aberrant repair mechanisms driven by alveolar epithelial cells II (AECII) associated with accelerated cellular senescence progressively ensure the endurance of pathological alterations and the development of self-perpetuating fibrosis [22]. Once pro-fibrotic factors are secreted, the differentiation and activation of myofibroblast, a heterogeneous cell population able to express contractile proteins, extracellular matrix proteins, and fibrogenic cytokines, lead to a devastating accumulation of the extracellular matrix (ECM) and tissue architecture distortion [23]. In addition to local fibroblast, some controversies still exist about the causative role of the epithelial–mesenchymal transition (EMT) into the genesis of myofibroblast precursors [24]. Interestingly, in a process defined as mesothelial–mesenchymal transition (MMT), the differentiation of pleural mesothelial cells into myofibroblast has been demonstrated [25]. Specifically, the authors reported that a TGF-mediated reduction and loss of Wilms’ tumor 1 expression combined to an upregulation of α-SMA may be crucial in MMT’s development [25]. 

In addition, despite that several involved pro-fibrotic factors have been identified, such as the platelet-derived growth factor (PDGF), the tumor necrosis factor (TNF), endothelin-1, the connective tissue growth factor (CTGF), and osteopontin, transforming growth factor beta-1 (TGF-β1) remains one of the most important molecular drivers for IPF [18]. By the activation of both SMAD-canonical and non-canonical signaling pathways, it is known to modulate the transcription of several mediators, ECM components, growth factors, the MMT involved in the development of fibrosis. Several kinases, such as mitogen-activated protein kinases (MAPKs), the extracellular signal-regulated kinase (ERK), and p38 kinase (p38 MAPK), have been involved in fibroblast proliferation and differentiation [26]. TGF-β1 is also involved in the preservation of myofibroblast by attenuating their apoptosis via the p-38 MAPK-PI3K-AKT pathway. Notably, an endothelin-1-mediated inhibition of fibroblast apoptosis involves the same molecular pathway, independently from TGF-β1 [27]. The interplay between TGF-β1 signaling and both Sonic hedgehog (Shh) and Wnt/β-catenin has been demonstrated supporting its role in EMT and fibrosis sustainment [28]. Evidence demonstrates that expression levels of Wnt proteins were significantly elevated in the lungs of patients with IPF compared to normal lungs [29,30,31]. Other mechanisms supporting the role of Wnt proteins in lung fibrosis development include the activation of myofibroblasts, the sustainment of cellular senescence, and promoting the pro-fibrotic phenotype of fibroblasts [31,32]. The Yes-associated protein (YAP) and the transcriptional coactivator with the PDZ-binding motif (TAZ), a core component of the Hyppo pathway, act as a sensor of cell mechanical force and have been reported to be activated in IPF lung fibroblasts, where they release pro-fibrotic factors and ECM proteins, resulting in an increase in ECM stiffness [33]. Alveolar macrophages represent another cellular population whose role seems to be relevant to lung fibrosis development. M2-like macrophage polarization, TLR2 signaling, apoptosis resistance, and TGF-β1 are some of the mechanisms activated in both resident and recruited macrophages [18,34,35,36]. 

Concentrations of PDGF have also been found to be increased in BALF and lung samples from patients with IPF [37]. PDGF, secreted by activated platelets and epithelial, inflammatory and surrounding mesenchymal cells, stimulates the proliferation of lung fibroblasts and the secretion of collagens and fibronectin through ERK1/2-dependent STAT1 activation [38]. CTGF is another important mediator of fibrosis, stimulating fibroblast proliferation and increasing extra-cellular matrix protein synthesis by fibroblasts. It seems that CTGF’s role depends on the concentrations of TGF-β, because CTGF functions as a downstream mediator of TGF-β action [39].

PF-ILD represents an umbrella term which includes different types of ILDs characterized by similar clinical behaviors but distinct driven mechanisms [40]. In CTD-ILDs, there is the production of self-reactive T and B cells, with the development of circulating autoantibodies leading to inflammation and organ damage. 

The prototype of fibrotic disease is undoubtedly represented by systemic sclerosis (SSc), whose lung involvement is often severe. The underlying mechanisms are different and not completely understood. The damage and apoptosis of endothelial cells (ECs), leading to perivascular inflammation, tissue hypoxia, and oxidative stress, are crucial in SSc pathogenesis [41]. Similar to IPF, TGF-β plays a key role in fibrosis development in SSc patients. The main pathogenetic event is characterized by small vessel vasculopathy due to autoantibody production. The autoantibodies cause a fibroblast dysfunction with a subsequent deposition of the extracellular matrix [8]. There is some accumulating evidence suggesting an association between autoantibodies and the development of PF-ILD, particularly anti-Scl-70 and anti-PM/SCL. Specifically, in patients who express pathogenetic antibodies, a baseline increase in TGF-β1 in fibroblasts is associated with high levels of Insulin-like growth factor II (IGF-2), which in turn leads to a rise of TGF-β2 and TGF-β3, as has been observed. These mechanisms finally contribute to a fibroblast-to-myofibroblast conversation, ECM protein collagen deposition, and fibronectin upregulation [42] Moreover, the abnormal expression of growth factors, such as PDGF1, PDGF2, and FGF, has been demonstrated in patients with SSc, resulting in fibroblast proliferation and aberrant ECM deposition [43,44,45]. 

Inflammation seems to be relevant in some non-IPF-ILDs such as hypersensitivity pneumonitis (HP) as well as rheumatoid arthritis (RA). In subjects with HP, the exposure to a wide variety of environmental antigen unleashes an immunological-mediated lung disease, resulting in a development of autoimmune features, fibroblast activation, and fibrosis. In a similar manner, in subjects with RA, an immune tolerance breakdown and an aberrant immune system activation, due to a potential cross-reaction between citrullinated proteins and similar antigens in the lungs, lead to the activation of inflammatory cells and T-lymphocytes. This heterogenous cellular infiltration and subsequent release of chemokines, growth factors, such as PDGF, and other cytokines, including IL-4, IL-13, and TGF-β finally lead to the proliferation of myofibroblasts and fibrosis [46]. Matrix metalloproteinases (MMPs) contribute to tissue remodeling pathways [47]. 

With an underlying genetic background, an aberrant amount of activated CD8+ T cells are also found in patients with myositis-associated interstitial lung diseases. Viral infection has been postulated to be a key triggering factor [48]. This may result in a granzyme B-mediated cleavage of several aminoacyl-tRNA synthetases (ARSs), including histidyl-tRNA synthetase, their transport from the lungs to lymph nodes, and the subsequent activation of helper T-cell and B-cell proliferation. As the autoantibodies recognize the autoantigen within the lungs, inflammation ignites and lung injury develops [48,49,50]. 

Lung stiffness represents a common hallmark of PF-ILDs and IPF. Interestingly, the expression of Lysyl oxidase family members (LOXL1 and LOXL2), which are key factors in ECM cross-linking, is increased in the lungs of IPF patients as well as other fibrotic ILDs, such as systemic sclerosis, where it has been demonstrated to directly contribute to extracellular matrix production and fibrosis [51,52]. Macrophages play a crucial role also in non-IPF ILDs. Studies show that the infiltration of CD163-positive macrophages into alveolar spaces is greater in fatal dermatomyositis-ILD than in survivors and increased serum CD163 levels are associated with a higher mortality rate in DM-ILD subjects [53,54]. 

## 3. Serum Biomarkers for Diagnosing and Monitoring Pulmonary Fibrosis 

A biomarker is an objectively measured characteristic that could be helpful for diagnosis, prognosis, or to evaluate the response to a therapeutic intervention. Although many serum biomarkers have been suggested for monitoring the progression of PPF, validation in prospective studies has not been obtained (Table 1). 

### 3.1. Idiopathic Pulmonary Fibrosis (IPF)

The clinical course of IPF remains difficult to predict, with some patients experiencing a slowly progressive disease over years and others experiencing rapid progression within months. 

A great deal of research has investigated the role of monocytes and neutrophils as potential biomarkers of progressive diseases. In these small studies, the monocyte levels were associated with an extension of the fibrosis during CT scans and with lung function decline [69,70]. Achaiah et al. found an association between monocyte levels and mortality; likewise, a strong association was observed between the neutrophil levels, lymphopenia, and the neutrophil–lymphocyte ratio (NLR) and a decline in the forced vital capacity (FVC) of patients with IPF [71]. 

Among the serum biomarkers, Krebs von den Lungen (KL-6) has been the most studied for assessing prognosis in all ILDs [75]. KL-6 is a high-molecular-weight glycoprotein, encoded by human mucin-1 (MUC1), and is predominantly expressed on the surface of alveolar type II pneumocytes and epithelial cells of the stomach, pancreas, and esophagus. This glycoprotein has pro-fibrotic and anti-apoptotic effects on lung fibroblasts [76,77]. Wakamatsu demonstrated a greater FVC decline in a retrospective cohort of 89 IPF patients with increased serum KL-6 levels (≥1000 U/mL) during follow-up compared with patients with no KL-6 increase [63]. Furthermore, baseline KL-6 levels were associated with acute-exacerbation (AE) risk and disease progression [63]. Similarly, in another research, KL-6 was associated with radiologic features of progression, including the extension of reticulation and honeycombing [78]. The role of KL-6 as a therapeutic biomarker during IPF acute exacerbations has been tested in AE-IPF patients treated with a high dose of corticosteroids; the survived patients presented a reduction in KL-6 levels [77]. Likewise, KL-6 might predict ILD patients who may benefit better from antifibrotic therapies [79]. 

Surfactant protein D (SP-D) is another protein associated with lung damage and fibrosis. SP-D might be a predictive indicator of the rate of decline in pulmonary function. Takahashi et al. documented high levels of SP-D in IPF patients experiencing more severe vital capacity (VC) and total lung capacity (TLC) decline [74]. As serum SP-D levels reflect alveolar epithelial dysfunction in IPF patients, SP-D serial measurements might be informative in clinical practice, providing response to treatment. Ikeda et al. found [80] a negative correlation between SP-D levels and lung function parameters during a 52-week follow-up period in response to pirfenidone therapy among IPF patients. In addition, matrix metalloproteinases (MMPs) are key regulator proteases involved in fibrosis pathogenesis, modulating extracellular matrix (ECM) degradation. MMP7 is one of the most promising prognostic biomarkers of IPF; MMP7 has been associated with mortality, and baseline concentrations were negatively correlated with DLCO [68]. Several studies marked a correlation between high MMP-7 levels, disease severity, and the risk of progression [65]. Longitudinally, MMP-7 assessment may help to discriminate patients with a higher risk of disease progression and lung function deterioration [81].

Likewise, periostin is upregulated during fibrotic responses, and baseline measurements may prognosticate the disease course [72]. CC-chemokine ligand 18 (CCL18) is another molecule produced by alveolar macrophages prompting the collagen production by lung fibroblasts; in patients with IPF, high baseline serum CCL18 concentrations (>50 ng/mL) were predictors of mortality and correlated with an acceleration of the disease [59]. Compared to KL-6 or SP-D, high CCL18 concentrations are more specific in defining the worsening of lung function [59].

In another research focusing on the role of prognostic biomarkers both of IPF and SSc-ILD, the authors found a significant association between IL-6 and a decline in gas exchange; no significant association was reported for IL-8, CCL2/MCP-1, IL-10, CXCL10/IP-10, the vascular endothelial growth factor (VEGF), the fibroblast growth factor (FGF)-2, and CX3CL1/fractalkine [62]. 

Lastly, contrasting results about VEGF arose from a different research documenting that VEGF levels could predict radiological and functional disease severity and progression [82]. According to a majority of studies, VEGF serum concentration increases as inflammation advances during IPF exacerbation, proving its role as a predictive disease progression biomarker that is helpful in the early identification of those patients who respond to antifibrotic therapy [82]. 

### 3.2. CTD-ILDs

As for IPF, a great deal of research has been conducted that focuses on the role of blood biomarkers in progressive fibrosing CTD-ILDs [83,84]. At this time, the clinical course of a CTD-ILD is highly variable, with some patients experiencing an accelerated loss of lung function, while others progress slowly or exhibit a stable disease [85,86]. 

Nagy et al. identified anti-SS-A antibodies as prognostic factors associated with a progressive disease [57]. Anti-SS-A antibodies, such as Ro52 and Ro60, are often useful for autoimmune disease diagnosis, and anti-SS-A/Ro52+ also has a prognostic value in SSc-ILD [57]. Previous small-cohort studies demonstrated that in anti-synthetase syndrome or inflammatory myopathy, patients who are anti-SS-A antibody positive develop more severe ILDs and are less responsive to immunosuppressive therapies [57]. The anti-Scl-70 antibodies are frequently present in patients with diffuse cutaneous SSc and may be associated with the faster progression of SSc-ILD. In particular, the presence of simultaneous anti-Scl-70 antibodies and anti-centromere-antibody absence indicates a faster decrease in FVC parameters and, therefore, an increased likelihood of progressive ILD, regardless of skin involvement, optimizing the timing of antifibrotic treatment initiation in patients with a high risk of progression [56,87,88].

Similarly, many other cytokines and growth factors have been associated with a higher risk of progression of SSc-ILD. 

In particular, high CCL18 baseline levels predict a >10% decline in FVC and a de novo development of extensive fibrosis, being associated with a lower survival rate [60,89,90,91]. In addition, in early SSc-ILD, CCL18 has been shown to be a potential surrogate of response to anti IL-6 therapy; SSc-ILD patients treated with tocilizumab who experienced a minor functional decline also documented a major reduction in CCL18 [61]. Another inflammatory marker, the C-reactive protein, could predict the long-term progression of SSc-ILD, and higher levels are associated with a rapid functional decline [92]. The Genetics Versus Environment in Scleroderma Outcome Study (GENISOS) study showed that CRP can predict mortality in SSc patients, resulting in an early identification of SSc-ILD patients who are more likely to experience worse clinical outcomes [91]. Likewise, the autoantibody anti-chemokine receptors CXCR3 and CXCR4 have been associated with progressive phenotype SSc-ILD. In particular, patients with a deterioration of lung function showed lower anti-CXCR3/4 antibody levels compared with those with a stable disease [55]. An immunosuppressive therapy-induced decrease in plasma CXCL4 levels, with larger declines during the first year of treatment, may serve as an effective biomarker for therapy efficacy [93].

As reported for IPF, IL-6 levels are significantly predictive of ILD progression/mortality [62]. KL-6 is a serum biomarker studied in several ILDs, including CTD-ILDs. In SSc-ILD patients, former small studies found that a higher KL-6 serum value (>500 U/mL) correlated negatively with lung function and positively with radiological impairment or the presence of extensive pulmonary fibrosis [94,95,96]. These recent findings were confirmed in a larger prospective cohort study, though no significant impact on mortality was reported [97], while contrasting results were described in older studies [64,98]. Finally, KL-6 was also studied as a therapeutic biomarker; in particular, a serum threshold of KL-6 levels of >2000 U/mL was able to differentiate patients with an unfavorable response to corticosteroids or cyclophosphamide [96,99,100]. Despite the fact that baseline KL-6 levels or their single measurement during the follow-up period cannot predict poor prognosis, KL-6 kinetic changes may be helpful in monitoring disease progression in SSc, with a reported cut-off of >193 U/mL [101].

Patients with RA can develop fibrosing ILD with rapid progression. Rheumatoid factor (RF), an autoantibody secreted by specialized B cells targeting the Fc region of IgG, contributes to the pathogenesis of RA by potentiating a cycle of immune-complex formation and complement fixation, which leads to additional autoantibody production [102]. Tyker et al. hypothesized a correlation between high-titer RF seropositivity and lung function decline or mortality [84]. The authors found that patients with the highest RF titers (≥60 IU/mL) have the greatest risk of disease progression and mortality, even after adjusting for the GAP score, a predictive mortality score which considers gender, age, and lung function and which is used in patients with IPF [103]. Previously, Nell et al. found an association between high-RF titers and a progression of erosive joint disease in RA [104]. Chen et al. showed that the baseline titers of RF were significantly higher in patients with progressive RA-ILD, and changes in serum levels of CXCL11 and MMP-13 over 5 years were significantly associated with the progression of RA-ILD [73]. Identifying RA-ILD patients with a progressive phenotype at an early stage can be facilitated through the identification of elevated anti-citrullinated peptide antibody (ACPA) levels, since its presence is associated with high morbidity and mortality [105]. In addition, KL-6 is a circulating marker that is also relevant for diagnosis and for progression in patients with RA-ILD. Retrospective studies showed that serum KL-6 levels were associated with ILD progression in patients with RA. Similar to IPF, in RA-ILD patients, KL-6 levels are associated with pulmonary functional (FVC, DLCO, and TLC) and exertional parameters (6 min waking distance). Likewise, the baseline KL-6 serum levels may predict patients with CTD-ILDs who are at higher risk of progression [65,106,107]. These data were corroborated in a further study on RA-ILD patients [108].

### 3.3. Other Fibrosing ILDs

HP is one of the most common ILDs. Sánchez-Dıez et al. showed elevated concentrations of serum KL-6 in fibrotic HP (fHP) patients as compared to non-fibrotic ones. Moreover, the concentrations of serum KL-6 displayed a negative association with TLC and DLCO [66]. Based on a large meta-analysis [109], HP patients have a higher expression of KL-6 compared to normal individuals, with a substantial increase in patients with fibrotic HP. Elevated KL-6 concentrations were found to be closely linked to the progression of ILD as well as a substantial functional decline. As mentioned above for IPF patients, SP-D is also prognostically useful in patients with fibrotic HP; in particular, high SP-D levels are correlated with a reduced survival [67]. Another useful biomarker for HP is C-C motif chemokine ligand 15 (CCL15), whose expression was increased in the lungs of fHP patients. In particular, higher CCL15 levels in bronchoalveolar lavage fluid (BALF) divided by BALF albumin (BALF CCL15/Alb) was significantly associated with a greater decline in pulmonary function over time [58].

In addition, HP patients whose disease progressed or who died had higher baseline YKL-40 levels than those who remained stable and survived [110]. Serum YKL-40 may be also a promising biomarker for monitoring disease activity and the development of fibrosis in patients with pulmonary sarcoidosis. Previously obtained data document a positive relation between serum YKL-40 and the ratio between carbon monoxide diffusion capacity and alveolar volume (DLCO/VA). In addition, serum YKL-40 levels are higher in patients with active sarcoidosis than those with inactive sarcoidosis and directly correlate with both sIL-2R and sACE levels in patients with active sarcoidosis, suggesting that YKL-40 is a marker for granuloma burden [111]. In sarcoidosis, the most well-established serum biomarker is the serum angiotensin-converting enzyme (sACE). High serum levels of ACE and IL2R, before treatment, correlate significantly with lung function improvement after 6 months of methotrexate treatment [112]. Lastly, CCL18, Chitotriosidase (CTO), and serum amyloid A (SAA) have the potential to be used as a marker to monitor disease and predict progression. CTO’s disease-prognostic role is demonstrated by its highest serum levels that are present in patients with progressive diseases and by its reduction resulting from prednisone or other immunosuppressant therapy [113,114,115,116]. 

Although the underlying molecular mechanisms are still not completely clear, anti-melanoma differentiation-associated gene 5 (MDA5)-positive dermatomyositis (MDA5+ DM) is reported to be associated with rapidly progressive ILD (RP-ILD), resulting in high mortality rates. However, significant disparities exist in the studied cohorts, and some MDA5+ DM patients did not develop RP-ILD [117]. In this respect, the other aforementioned molecules, such as KL-6, SP-D, and the Anti-Ro52 antibody, may be considered as prognostic biomarkers for these patients [118]. Although not specific to this disease, several blood-based biomarkers, such as serum ferritin, YKL-40, neopterin, KL-6, SP-D, IL18, and IFN-α, as well as antibody biomarkers, such as the Anti-Ro52 antibody or autoantibodies, and the circulating immune cell biomarker CD3+T cell can aid in the early diagnosis, prognosis, and treatment efficacy of DM-RPILD. Serum KL-6 is a specific biomarker for ILD severity and progression in MDA5+DM patients, since elevated KL-6 levels in the first four weeks of immunosuppressive treatment in MDA5+DM-ILD patients may indicate the occurrence of intractable RPILD. Serum IL-18 levels decrease after treatment, indicating that IL-18 levels can reflect a response to RPILD treatment in MDA5+DM patients [118].

## 4. Therapeutic Strategies in PPF

### 4.1. Idiopathic Pulmonary Fibrosis (IPF)

Several drugs have been developed against these abnormal fibrotic pathways (Table 2). Presently, no curative pharmacological therapy is available for IPF; however, two antifibrotic drugs (nintedanib and pirfenidone) are approved and licensed for IPF treatment, as they reduce the pace of functional decline as well the progression of fibrosis, limiting acute exacerbations and influencing overall mortality [119,120,121,122,123]. While the efficacy of these antifibrotic agents has been established and they are currently recommended in guidelines, several other molecules are under investigation or are in the final stages of research. 

Phosphodiesterase 4B (PDE4B) is part of the family of PDE4 inhibitors, which regulates the production of pro- and anti-inflammatory cytokines via cAMP degradation. BI 1015550 is an oral preferential inhibitor of PDE4B. BI1015550 demonstrated anti-inflammatory activity, inhibiting TNF-α and IL-2 in human peripheral blood mononuclear cells. In addition, in lung fibroblasts from patients with IPF, BI 1015550 inhibited TGF-β1-stimulated myofibroblast transformation, IL-1β-induced cell proliferation, the mRNA expression of extracellular matrix proteins, as well as the fibroblast growth factor [124]. Therefore, co-administration with the antifibrotic nintedanib appeared to be synergistic, while co-administration with pirfenidone did not appear to have additional inhibitory effects in vitro [124]. More recently, a multicenter, randomized, double-blind, phase-2 trial was conducted in patients with IPF. The primary endpoint was a change from the baseline in FVC following 12 weeks of treatment. Among patients without previous antifibrotic use, the FVC median change was +5.7 mL in the group treated with BI1015551, compared with −81.7 mL in the placebo group, and in the same way, the FVC median change was +2.7 mL in the treated group and −59 mL in the placebo group in patients who were already treated with nintedanib or pirfenidone [125]. In the subsequent and ongoing phase-3 study (FIBRONEER-IPF trial) (NCT05321069), patients with IPF are being randomized 1:1:1 to receive 9 mg or 18 mg of BI 1015550 or a placebo two times per day alone or in combination with the current antifibrotic standard of care. The primary endpoint is the absolute change in FVC at week 52. Actually, 1177 participants are enrolled, and it is estimated that this study will be concluded within November 2024 [126]. 

Moreover, treprostinil, a prostacyclin mimetic analogue that also acts on prostaglandin receptors, in particular, on prostaglandin E receptor 2 (EP2) and prostaglandin D receptor 1 (DP1), is under evaluation for IPF. In vivo studies on EP2 and DP1 found that treprostinil administration resulted in reduced fibroblast proliferation, collagen secretion, and fibroblast-to-myofibroblast differentiation [127]. Inhaled treprostinil was related with advantages limiting FVC deterioration and the acute exacerbations of underlying lung diseases in a phase-3 study—the INCREASE trial—in patients with precapillary pulmonary hypertension due to ILDs, including IPF. These promising results, coupled with data of preclinical models about the antifibrotic activity of treprostinil, have prompted a further investigation into IPF [128]. TETON is the first phase-3 randomized clinical trial (RCT) (NCT04708782) evaluating inhaled treprostinil in IPF patients [129]; this study will be concluded within June 2025, and results will be available by 2026. 

Fibrotic diseases can be mediated by lysophosphatidic acid (LPA), which signals via six LPA receptors (LPA1–6). BMS 986278 is a high-affinity small-molecule antagonist of LPA1. A phase-2 trial (NCT04308681) showed that 600 mg of BMS-986020 BID significantly reduced the rate of decline in FVC from the baseline to week 26 when compared with a placebo in patients with IPF [130]. Autotaxin is the enzyme responsible for LPA production. 

Two phase-3 RCTs, ISABELA 1 and ISABELA 2, assessed the efficacy and safety of the autotaxin inhibitor (GLPG1690), ziritaxestat, in patients with IPF. The trials were terminated early because of an increased mortality in the ziritaxestat group, and the drug did not improve clinical outcomes compared with the placebo in patients with IPF receiving the standard of care treatment with pirfenidone or nintedanib or in those not receiving the standard of care treatment [131]. Possible explanations for the failure of the ISABELA trial include the interaction with underlying antifibrotic therapies, possibly leading to a significant increase in nintedanib levels with subsequential unpredicted side effects. In the previous phase-2a study, ziritaxestat was administered as a monotherapy. 

Another prematurely interrupted RCT was the randomized, double-blind, placebo-controlled, multicenter phase-3 ZEPHYRUS trial, which aimed to assess the efficacy and safety of pamrevlumab—a connective-tissue-growth-factor (CTGF) inhibitor in patients with IPF. The primary endpoint was a change from the baseline for FVC at week 48. The results show a mean decline in FVC from the baseline to week 48 of 260 mL in the pamrevlumab arm and of 330 mL in the placebo arm. Furthermore, this study did not meet the secondary endpoint. Based on these results, the clinical trial was stopped [132].

Pentraxin-2 (PTX2) inhibits the expression of TGF-B, the central fibrosis mediator, and the differentiation of monocytes into pro-fibrotic macrophages and fibrocytes. Despite promising results in terms of safety and efficacy arising from the preliminary phase 2 trial [133], the intravenous infusion of recombinant human pentraxin-2 (rhPTX-2) failed to meet the primary endpoint in the phase-3 trial [134]. 

### 4.2. CTD-ILDs

Therapies for patients with CTD-ILDs are currently based on immunosuppressive agents, although their efficacy in slowing disease progression is not fully elucidated, and the quality of evidence for some disorders is not remarkable. Therefore, the antifibrotic agents already approved for IPF treatment have been tested in RCTs in patients with CTD-ILDs (Table 3).

The INBUILD study investigated the efficacy of nintedanib in patients with progressive phenotype F-ILDs, including CTD-ILD patients [135]. Eighty-two patients in the trial had a CTD-ILD (mainly, RA-ILD and SSc-ILD). this study documented a reduction in the annual rate of FVC decline, and, therefore, nintedanib was licensed for progressive phenotype F-ILDs. Similar results were reported in the Safety and Efficacy of Nintedanib in Systemic SClerosIS (SENSCIS) trial, showing the impact of nintedanib on SSc-ILD, leading to regulatory agencies approving this medication as an additional treatment for this disorder [136]. 

Likewise, the efficacy of pirfenidone was also evaluated in SSc-ILD and RA-ILD. The randomized, double-blind, placebo-controlled, phase-2 trial, TRAIL1, assessed the safety, tolerability, and efficacy of pirfenidone for the treatment of patients with RA-ILD. This trial was stopped early due to slow recruitment and did not reach the primary end point (≥10% decline in FVC% was predicted or a death over 52 weeks), but the rate of decline in FVC in the pirfenidone group was −66 mL/year, compared with −146 mL/year in the placebo group, a relative reduction of 55% [137]. The efficacy of pirfenidone as an add-on therapy in SSc-ILD has been reported in several case reports and retrospective studies [138,139,140,141]. However, a study conducted on SSc-ILD found no significant difference in the efficacy of pirfenidone in improving or stabilizing lung functions [142]. Subsequently, Scleroderma Lung Study III demonstrated that the combination of mycophenolate and pirfenidone led to a more rapid improvement of FVC, though an increase in adverse side effects was reported [143]. Therefore, altogether, these data do not support the use of pirfenidone for non-IPF ILDs. 

In the pre-antifibrotic era, immunosuppression was long considered the cornerstone of CTD-ILD treatment with two main purposes: ILD-onset prevention and the deceleration of ILD progression once existent. In RA, an historical debate about the role of methotrexate (MTX), which is considered to be highly effective for articular involvement but with potential lung toxicity, has been reappraised based on large data documenting a beneficial effect of MTX in preventing RA-ILD [144], reducing RA-ILD progression [145], and improving RA-ILD mortality [146]. Similarly, a protective role of abatacept (Aba)—a T-cell-activation inhibitor—and rituximab (RTX)—an anti-CD20 chimeric monoclonal antibody—in ILD progression has been postulated. Very interestingly, a recent metanalysis of 10 phase-3 clinical trials suggests that the combination of MTX and Aba might be complementary in limiting ILD development. The magnitude of this effect was higher in younger subjects without a smoking history, high disease activity (DAS28), and RF or ACPA positivity and with no prior TNF-inhibitor or corticosteroid use. At the same time, there is strong evidence about other biological and targeted synthetic disease-modifying antirheumatic drugs (b/t sDMARDs), and a recent metanalysis suggests that tofacitinib—a novel JAK-inhibitor—might play a major preventive role in preventing ILD among RA patients [147]. 

SSc-ILD treatment is also based on immunosuppressors that are based on high quality RCTs. Originally, CYC was tested versus a placebo in RCTs, confirming a benefit in terms of FVC and TLC, as well some patient-reported outcomes—including dyspnea—despite a major risk of adverse events/toxicities [148]. Subsequently, in Scleroderma Lung Study II, MMF showed non-inferiority versus CYC with a favorable safety profile. Both studies confirmed that FVC improvement remains stable at 24 months [149]. Other more recent evidence also emerged from other immunosuppressive agents. The INSIST Trial, a single-center, open-label, prospective, randomized, controlled pilot study, compared the efficacy of tacrolimus versus MMF in patients with progressive SSc-ILD. The primary endpoint was the difference in change in the FVC rate at 24 weeks; secondary outcomes included an absolute change in FVC, skin scores, and 6MWD. Tacrolimus produced a comparable improvement of MMF in all primary and secondary endpoints, with a favorable safety profile in patients with SSc-ILD [150]. The focuSSced trial, a multicenter, double-blind, placebo-controlled, phase-3 trial, suggests that TCZ may have the potential to preserve lung function in patients with early diffuse SSc-ILD and elevated acute-phase reactants [151]. More limited evidence of lower quality is present for other CTD-ILDs.

The results of the abovementioned studies have led to a recent update of the American College of Rheumatology (ACR) Guideline for the Treatment of Interstitial Lung Disease in People with Systemic Autoimmune Rheumatic Disease (SARD), despite the fact that a full text of this manuscript has still not been published. The summary of this guideline provides evidence-based recommendations for first-line ILD treatments, the treatment for ILD progression despite first-line treatment, and the treatment of rapidly progressive ILD (RP-ILD) [152]. In particular, for patients with SSc-ILD, either MMF, TCZ, or RTX is recommended as a first-line option, with a strong recommendation against the use of glucocorticosteroids (GCs). Conversely, MMF, AZA, and RTX are potential first-line treatments for RA-ILD, mixed CTD-ILD, and Sjogren’s-associated ILD, whereas in these cases, a short-course of GCs can be associated. Additional second-line therapies—including CYC, AZA, TCZ, and JAK-i—can be considered in all the mentioned CTD-ILDs, whereas these are not included as first-line strategies. Particularly complex is the pharmacological management of myositis-associated ILDs, because of the substantial heterogeneity of their clinical courses and the lack of evidence-based guidelines. Recent studies have highlighted the importance of evaluating myositis-specific autoantibodies, in particular, the anti-melanoma differentiation-associated gene 5 (MDA5) and anti-aminoacyl tRNA synthetase (ARS) antibodies, in order to evaluate clinical phenotypes and treatment choices. The anti-MDA5 antibody phenotype is associated with rapidly progressive ILD with a poor prognosis. These patients require immediate combined immunosuppressive treatment with high-dose glucocorticoids and calcineurin inhibitors (CNIs). Patients with rapidly progressive ILD should be treated with a more aggressive triple-combination therapy of high-dose glucocorticoids, CNIs, and intravenous cyclophosphamide. In patients with refractory ILDs despite adequate treatments, including triple-combination therapies, additional salvage therapies (such as rituximab, tofacitinib, and plasma exchange) should be evaluated. Patients with ILD and anti-ARS antibodies respond better to glucocorticoid treatment, but with frequent relapses. Therefore, treatment with glucocorticoids and immunosuppressants is often necessary to achieve favorable long-term disease control. Randomized controlled trials are needed to resolve clinical questions such as which immunosuppressant is most suitable for initial induction therapy or long-term maintenance therapy in patients with idiopathic inflammatory myopathies [153]. Nintedanib can be used in other CTD-ILDs, according to the INBUILD criteria. As per IPF, the PDE-4b inhibitor—BI 1015550—is under current investigation in PPFs—including CTD-ILDs—in the study NCT05321082. Results are to be available by 2025. 

### 4.3. Sarcoidosis

The treatment of sarcoidosis is currently indicated in the case of extensive ILD or pulmonary fibrosis or when it involves critical organs, especially the eyes and heart. Glucocorticoid treatment with 20 mg of prednisone once a day remains the cornerstone of treatment. For patients with symptomatic pulmonary sarcoidosis who have been treated with glucocorticoids and have continued disease or unacceptable side-effects from glucocorticoids, the addition of methotrexate, azathioprine, Leflunomide, mycophenolate mofetil, or Hydroxychloroquine has been suggested. Patients treated with glucocorticoids and other immunosuppressive agents and who have progressive pulmonary disease or extrapulmonary manifestations (cardiac and CNS), the addition of infliximab or adalimumab could be considered [154]. Pulmonary sarcoidosis can progress to fibrosis in approximately 5% of patients, leading to a decline in lung function, respiratory failure, and death. The efficacy of antifibrotic agents in sarcoidosis with fibrosis is an area of ongoing research. The efficacy of pirfenidone in fibrotic sarcoidosis is being studied in a clinical trial [155], while nintedanib is licensed for PPF [135]. In recent years, there have been several early-phase trials of novel therapeutic agents in fibrotic pulmonary sarcoidosis. NCT05415137 [156] is the most advanced ongoing drug trial in pulmonary sarcoidosis. This multicenter randomized, double-blind, placebo-controlled phase-3 study will evaluate the safety and efficacy of two IV doses of Efzofitimod (a novel fusion protein immunomodulator that selectively binds the immunoregulatory receptor Neuropilin-2) given every 4 weeks to patients with pulmonary sarcoidosis who receive stable doses of oral corticosteroids taken with or without an additional immunosuppressant therapy. Likewise, NCT04064242 [157] is a multi-national randomized, double-blind, placebo-controlled phase-2 study evaluating the safety, efficacy, and tolerability of CMK389 (a fully human IgG1 monoclonal antibody directed against IL-18) given every 4 weeks for 16 weeks in patients with chronic pulmonary sarcoidosis. Finally, NCT05314517 [158] is a randomized, double-blind, placebo-controlled phase-2 trial with an open-label extension evaluating the safety and efficacy of Namilumab (a fully human IgG1 monoclonal anti-GM-CSF antibody) given every 4 weeks for a total of 26 weeks, followed by an optional 28 weeks in patients with chronic pulmonary sarcoidosis. The results of the abovementioned RCTs will add novel information to the therapeutic landscape of sarcoidosis. 

### 4.4. Fibrotic HP

For hypersensitivity pneumonitis (HP), exposure assessment and antigen avoidance are critical for the management of these patients. The latest guidelines for the diagnosis of HP propose that patients be categorized as having non-fibrotic (purely inflammatory) HP or fibrotic HP (mixed inflammatory and fibrotic or purely fibrotic) [159]. 

For non-fibrotic HP, the first approach is steroid therapy (such as prednisolone) with slow tapering at the minimum useful dosage, while the evidence basis for the use of immunomodulators is poor. Some retrospective analyses have shown an improvement in lung function after a year of treatment with MMF or azathioprine [160,161,162]. However, another retrospective study found no difference in lung function decrease or survival between patients treated with azathioprine or MMF plus prednisone or only prednisone [163]. A retrospective study of 20 patients showed that treatment with rituximab for 6 months led to the stabilization or improvement of FVC and DLCO in patients with HP whose disease had not improved following antigen avoidance and corticosteroid therapy [164]. Immunosuppression is also commonly used in the treatment of fibrotic HP but has not been shown to slow the progression of this disease. Despite future RCTs being required to confirm its efficacy, both immunomodulant agents AZA and MMF were tested for fibrotic HP. Terras et al. [162] demonstrated that the oral administration of AZA for 2 years is associated with a significant improvement in FVC and a slight but nonstatistical increase in DLCO and 6MWT. Similar results in improving lung function as assessed for DLCO were documented using both MMF or AZA for 1 year [160].

Antifibrotic therapy should be considered in patients with progressive fibrosing ILDs, as in the INBUILD study, wherein 173 patients with fibrotic HP were included [135]. Likewise, pirfenidone was investigated as a treatment for fibrotic HP. Unfortunately, the phase-3 trial was underpowered due to the COVID-19 pandemic and did not meet the primary endpoint (rate of FVC decline at 52 weeks). However, improvements in progression free survival (PFS) and significant impacts on FVC decline at 26 weeks have been reported [123]. These data corroborate another study on pirfenidone in PPF—the RELIEF study. While this study was prematurely terminated due to futility and slow enrollment, an analysis of the data from the 127 patients that enrolled, of whom 57 had fibrotic HP, demonstrated a smaller decline in FVC% as predicted over 48 weeks in patients who received pirfenidone versus a placebo [165]. 

## 5. Conclusions

PF-ILDs represent a heterogenous family of diffuse lung conditions characterized by progressive lung function deterioration and a reduced quality of life due to lung fibrosing alterations. The therapeutic scenario has witnessed promising steps forward for patients affected by PF-ILDs. However, outcomes differ among patients. In this respect, novel biomarkers for the prompt recognition of progressive phenotypes as well as for predicting a positive response to treatment are required. 

## Figures and Tables

**Table 1:** Serum biomarkers of pulmonary fibrosis. CCL15: Chemokine C-C motif ligand 15; CCL18: Chemokine (C-C motif) ligand 18; CHP: chronic hypersensitivity pneumonitis; CTDs: Connective tissue disorders; CXCR3: C-X-C chemokine receptors 3; CXCR4: C-X-C chemokine receptors 4; DLCO: diffusing capacity for carbon monoxide; FVC; forced vital capacity; KL-6: Krebs von den Lungen 6; IL-6: Interleukin-6; ILD: interstitial lung disease; IPAF: interstitial pneumonia with autoimmune features; IPF: idiopathic pulmonary fibrosis; RA: rheumatoid arthritis; SSc: systemic sclerosis; TLC: total lung capacity.

Biomarker	Population	Findings	References
Anti-CXCR3/CXCR4	SSc (n = 327)	○ Patients with a deterioration of lung function show lower anti-CXCR3/4 ab levels compared with those with stable disease.	Weigold et al. [55]
Anti-Scl 70	SSc (n = 117)	○ Presence of anti-Scl 70 is a significant predictor of ILD.	Wangkaew et al. [56]
Anti-SS-A/Ro52+	CTD/IPAF (n = 107)	○ Anti-SS-A is associated with rapid ILD progression.	Nagy et al. [57]
CCL15	HP (n = 51)	○ Serum CCL15 is significantly (*p* = 0.003) higher in CHP patients (29.1 ± 2.1 μg/mL) than in IPF patients (19.7 ± 1.3 μg/mL; *p* = 0.01) and healthy controls (19.5 ± 1.7 μg/mL).	Watanabe [58]
CCL18	IPF (n = 72)	○ Baseline concentration correlates with an acceleration of the disease;	Prasse et al. [59]
	SSc (n = 83)		Tiev et al. [60]
	SSc (n = 87)	○ High baseline serum CCL18 concentrations (>50 ng/mL) are predictors of mortality;	Khanna et al. [61]
		○ CCL18 is a predictive biomarker of lung disease worsening;	
		○ Potential surrogate of response to anti IL-6 therapies.	
IL-6	SSc (n = 286)	○ Serum IL-6 was significantly predictive of DLCO deterioration.	De Lauretis [62]
KL-6	IPF (n = 89)	○ Higher FVC% predicted a decline with serum KL-6 levels (≥1000 U/mL);	Wakamatsu et al. [63]
	Early-SSc (n = 50)	○ KL-6 value of 1273 U/mL discriminates patients who developed ESLD;	Kuwana et al. [64]
	RA-ILD (n = 84)	○ A high KL-6 level (≥685 U/mL) was an independent prognostic factor for mortality (hazard ratio [HR]: 2.984; *p* = 0.016);	Kim et al. [65]
	HP (n = 49)	○ Serum KL-6 levels correlated negatively with TLC (r = −0.485; *p* = 0.0103) at 12 months; ○ Serum KL-6 levels correlated negatively with DLCO (r = −0.534; *p* = 0.0002) at 12 months;	Sánchez-Díez [66]
	f-HP (n = 185)	○ High SP-D levels correlated with a reduced survival.	Ejima et al. [67]
MMP7	IPF (n = 97)	○ Baseline concentrations positively associated with mortality; ○ Baseline concentrations were negatively correlated with DLCO.	Tzouvelekis et al. [68]
Monocytes	IPF (n = 37)	○ Monocyte levels correlate positively with the extent of fibrosis.	Fraser et al. [69]
	IPF (n = 2067)	○ A monocyte count of >0.60 × 109 cells/L is associated with IPF progression, all-cause hospitalization, and all-cause mortality over 1 year.	Kreuter et al. [70]
	IPF (n = 128)	○ A change in monocyte count of >0.50 × 109 cells/L from baseline is significantly associated with all-cause hospitalization; ○ A monocyte count of >0.90 × 109 cells/L is associated with all-cause mortality	Achaiah et al. [71]
Periostin	IPF (n = 54)	○ Upregulated during fibrotic responses; ○ Baseline measurement may prognosticate the disease course.	Naik et al. [72]
RF	RA (n = 60)	○ Baseline serum RF is significantly higher in patients with progressive RA-ILD (*p* < 0.05).	Chen et al. [73]
SP-D	IPF (n = 52)	○ Both SP-A and SP-D concentrations are significantly correlated with the extent of alveolitis (reversible change);	Takahasi et al. [74]
		○ No correlation with the progression of fibrosis (irreversible change);	
		○ SP-D concentration is related to the extent of parenchymal collapse and the rate of deterioration per year in pulmonary function.	

**Table 2:** Therapeutic strategies of IPF. CTGF: connective tissue growth factor; DP_1_: Prostaglandin D1; EP_2_: Prostaglandin E2; FGF: fibroblast growth factor; FGFR: FGF receptor; IL-2: Interleukin 2; IL-4: Interleukin 4; IL-13: Interleukin 13; LPA: A lysophosphatidic acid; LPAR_1:_ lysophosphatidic acid receptor 1; MMPs: matrix metalloproteinases; PDE4B: phosphodiesterase 4B; PDGF: platelet-derived growth factor; PDGFR: PDGF receptor; PPAR: Peroxisome proliferator-activated receptor; rhPTX-2: recombinant human pentraxin-2; TGF-β: transforming growth factor beta; TNF-α: tumor necrosis factor α; VGFR: vascular endothelial growth factor receptor.

Molecule	Route of Administration	Mechanism of Action	Key Trials	Status
BI 1015550	Oral	↓ PDE4b ↓ TNF-α ↓ IL-2 ↓ FGF ↓ myofibroblast transformation	FIBRONEER-IPF NCT05321069 NCT04419506 (Phase 2)	Under evaluation
BMS 986278	Oral	LPAR_1_ antagonist ↓ myofibroblast activation	NCT04308681—Phase 2	Under evaluation
Pirfenidone	Oral	↓ TGF-β ↓ PDGF ↓ TNF-α ↓ IL-13 ↓ IL-4 ↑ MMPs	ASCEND—NCT01366209 Capacity 1—NCT00287729 Capacity 2—NCT00287716 SP3—Japanese	Approved
Nintedanib	Oral	FGFR inhibitor PDGFR inhibitor VGFR inhibitor	INPULSIS-1—NCT01335464 INPULSIS-2—NCT01335477	Approved
Pamrevlumab	Intravenous	↓ CTGF	ZEPHYRUS 2—NCT04419558	Not approved
PRM-151 (rhPTX-2)	Intravenous	↓ Inflammation ↓ Fibrosis	WA42404—NCT02550873 STARSCAPE—NCT04552899	Not approved
Treprostinil	Inhaled	↑ EP_2_ ↑ DP_1_ ↑ PPAR ↓ Cell proliferation ↓ Collagen synthesis ↓ Inflammation ↓ Fibroblast proliferation	INCREASE—NCT02630316 TETON—NCT04708782	Under evaluation
Ziritaxestat	Oral	↓ Autotaxin ↓ LPA	ISABELLA 1—NCT03711162 ISABELLA 2—NCT03733444	Not approved

**Table 3:** Add-on options for treatment of CTD-ILDs. CTD: connective tissue disease; ILD: interstitial lung disease; MCTD: Mixed connective tissue disease; PDE4bi: phosphodiesterase 4 inhibitor; RA-ILD: rheumatoid arthritis—ILD; SSc-ILD: scleroderma-associated—ILD.

RA-ILD			
	Molecule	Key Trials	Recommendation
	BI 1015550 (PDE4Bi)	NCT05321082	Under evaluation as per PPF
	Nintedanib	INBUILD—NCT02999178	Recommended for ILD progression despite first-line treatment
	Pirfenidone	TRAIL 1—NCT02808871	Not recommended
**SSc-ILD**			
	**Molecule**	**Key Trials**	**Recommendation**
	BI 1015550 (PDE4Bi)	NCT05321082	Under evaluation as per PPF
	Nintedanib	INBUILD—NCT02999178 SENSCIS—NCT02597933	Recommended as additional first-line ILD option
	Pirfenidone	NCT03856853 SLSIII—NCT03221257	Not recommended
	Tacrolimus	INSIST—CTRI/2021/11/037864	Under evaluation
	Tocilizumab	focuSSced—NCT02453256	Recommended as first-line preferred ILD option
**Other CTD-ILDs (Myositis, MCTD, Sjogren)**			
	**Molecule**	**Key Trial**	**Recommendation**
	BI 1015550 (PDE4Bi)	NCT05321082	Under evaluation as per PPF
	Nintedanib	INBUILD—NCT02999178	Recommended for ILD progression despite first-line treatment

## Data Availability

The data are openly available.

## References

[1] Travis W.D., Costabel U., Hansell D.M., King T.E.J., Lynch D.A., Nicholson A.G., Ryerson C.J., Ryu J.H., Selman M., Wells A.U. (2013). An Official American Thoracic Society/European Respiratory Society Statement: Update of the International Multidisciplinary Classification of the Idiopathic Interstitial Pneumonias. Am. J. Respir. Crit. Care Med..

[2] Valeyre D., Freynet O., Dion G., Bouvry D., Annesi-Maesano I., Nunes H. (2010). [Epidemiology of interstitial lung diseases]. Presse Med..

[3] Piciucchi S., Tomassetti S., Ravaglia C., Gurioli C., Gurioli C., Dubini A., Carloni A., Chilosi M., Colby T.V., Poletti V. (2016). From “Traction Bronchiectasis” to Honeycombing in Idiopathic Pulmonary Fibrosis: A Spectrum of Bronchiolar Remodeling Also in Radiology?. BMC Pulm. Med..

[4] Raghu G., Remy-Jardin M., Richeldi L., Thomson C.C., Inoue Y., Johkoh T., Kreuter M., Lynch D.A., Maher T.M., Martinez F.J. (2022). Idiopathic Pulmonary Fibrosis (an Update) and Progressive Pulmonary Fibrosis in Adults: An Official ATS/ERS/JRS/ALAT Clinical Practice Guideline. Am. J. Respir. Crit. Care Med..

[5] Khine N., Mudawi D., Rivera-Ortega P., Leonard C., Chaudhuri N., Margaritopoulos G.A. (2020). Rapidly Non-Ipf Progressive Fibrosing Interstitial Lung Disease: A Phenotype with an Ipf-like Behavior. Sarcoidosis Vasc. Diffus. Lung Dis. Off. J. WASOG.

[6] Olson A.L., Gifford A.H., Inase N., Fernández Pérez E.R., Suda T. (2018). The Epidemiology of Idiopathic Pulmonary Fibrosis and Interstitial Lung Diseases at Risk of a Progressive-Fibrosing Phenotype. Eur. Respir. Rev. Off. J. Eur. Respir. Soc..

[7] Hoffmann-Vold A.-M., Weigt S.S., Saggar R., Palchevskiy V., Volkmann E.R., Liang L.L., Ross D., Ardehali A., Lynch J.P., Belperio J.A. (2019). Endotype-Phenotyping May Predict a Treatment Response in Progressive Fibrosing Interstitial Lung Disease. EBioMedicine.

[8] Wong A.W., Ryerson C.J., Guler S.A. (2020). Progression of Fibrosing Interstitial Lung Disease. Respir. Res..

[9] Maher T.M., Wuyts W. (2019). Management of Fibrosing Interstitial Lung Diseases. Adv. Ther..

[10] Cottin V., Hirani N.A., Hotchkin D.L., Nambiar A.M., Ogura T., Otaola M., Skowasch D., Park J.S., Poonyagariyagorn H.K., Wuyts W. (2018). Presentation, Diagnosis and Clinical Course of the Spectrum of Progressive-Fibrosing Interstitial Lung Diseases. Eur. Respir. Rev. Off. J. Eur. Respir. Soc..

[11] Nalysnyk L., Cid-Ruzafa J., Rotella P., Esser D. (2012). Incidence and Prevalence of Idiopathic Pulmonary Fibrosis: Review of the Literature. Eur. Respir. Rev. Off. J. Eur. Respir. Soc..

[12] Ley B., Collard H.R., King T.E.J. (2011). Clinical Course and Prediction of Survival in Idiopathic Pulmonary Fibrosis. Am. J. Respir. Crit. Care Med..

[13] Hambly N., Farooqi M.M., Dvorkin-Gheva A., Donohoe K., Garlick K., Scallan C., Chong S.G., MacIsaac S., Assayag D., Johannson K.A. (2022). Prevalence and Characteristics of Progressive Fibrosing Interstitial Lung Disease in a Prospective Registry. Eur. Respir. J..

[14] Cottin V., Teague R., Nicholson L., Langham S., Baldwin M. (2022). The Burden of Progressive-Fibrosing Interstitial Lung Diseases. Front. Med..

[15] Nasser M., Larrieu S., Si-Mohamed S., Ahmad K., Boussel L., Brevet M., Chalabreysse L., Fabre C., Marque S., Revel D. (2021). Progressive Fibrosing Interstitial Lung Disease: A Clinical Cohort (the PROGRESS Study). Eur. Respir. J..

[16] Faverio P., Piluso M., De Giacomi F., Della Zoppa M., Cassandro R., Harari S., Luppi F., Pesci A. (2020). Progressive Fibrosing Interstitial Lung Diseases: Prevalence and Characterization in Two Italian Referral Centers. Respiration.

[17] Ryerson C.J., Vittinghoff E., Ley B., Lee J.S., Mooney J.J., Jones K.D., Elicker B.M., Wolters P.J., Koth L.L., King T.E.J. (2014). Predicting Survival across Chronic Interstitial Lung Disease: The ILD-GAP Model. Chest.

[18] Samarelli A.V., Tonelli R., Marchioni A., Bruzzi G., Gozzi F., Andrisani D., Castaniere I., Manicardi L., Moretti A., Tabbì L. (2021). Fibrotic Idiopathic Interstitial Lung Disease: The Molecular and Cellular Key Players. Int. J. Mol. Sci..

[19] Nyunoya T., Monick M.M., Klingelhutz A., Yarovinsky T.O., Cagley J.R., Hunninghake G.W. (2006). Cigarette Smoke Induces Cellular Senescence. Am. J. Respir. Cell Mol. Biol..

[20] Bloom S.I., Islam M.T., Lesniewski L.A., Donato A.J. (2023). Mechanisms and Consequences of Endothelial Cell Senescence. Nat. Rev. Cardiol..

[21] Schulz L., Hornung F., Häder A., Radosa L., Brakhage A.A., Löffler B., Deinhardt-Emmer S. (2023). Influenza Virus-Induced Paracrine Cellular Senescence of the Lung Contributes to Enhanced Viral Load. Aging Dis..

[22] Albera C., Verri G., Sciarrone F., Sitia E., Mangiapia M., Solidoro P. (2021). Progressive Fibrosing Interstitial Lung Diseases: A Current Perspective. Biomedicines.

[23] Phan S.H. (2008). Biology of Fibroblasts and Myofibroblasts. Proc. Am. Thorac. Soc..

[24] Hinz B. (2012). Mechanical Aspects of Lung Fibrosis: A Spotlight on the Myofibroblast. Proc. Am. Thorac. Soc..

[25] Karki S., Surolia R., Hock T.D., Guroji P., Zolak J.S., Duggal R., Ye T., Thannickal V.J., Antony V.B. (2014). Wilms’ Tumor 1 (Wt1) Regulates Pleural Mesothelial Cell Plasticity and Transition into Myofibroblasts in Idiopathic Pulmonary Fibrosis. FASEB J. Off. Publ. Fed. Am. Soc. Exp. Biol..

[26] Xiao L., Du Y., Shen Y., He Y., Zhao H., Li Z. (2012). TGF-Beta 1 Induced Fibroblast Proliferation Is Mediated by the FGF-2/ERK Pathway. Front. Biosci. Landmark Ed..

[27] Kulasekaran P., Scavone C.A., Rogers D.S., Arenberg D.A., Thannickal V.J., Horowitz J.C. (2009). Endothelin-1 and Transforming Growth Factor-Beta1 Independently Induce Fibroblast Resistance to Apoptosis via AKT Activation. Am. J. Respir. Cell Mol. Biol..

[28] Zhou B., Liu Y., Kahn M., Ann D.K., Han A., Wang H., Nguyen C., Flodby P., Zhong Q., Krishnaveni M.S. (2012). Interactions between β-Catenin and Transforming Growth Factor-β Signaling Pathways Mediate Epithelial-Mesenchymal Transition and Are Dependent on the Transcriptional Co-Activator CAMP-Response Element-Binding Protein (CREB)-Binding Protein (CBP). J. Biol. Chem..

[29] Königshoff M., Balsara N., Pfaff E.-M., Kramer M., Chrobak I., Seeger W., Eickelberg O. (2008). Functional Wnt Signaling Is Increased in Idiopathic Pulmonary Fibrosis. PLoS ONE.

[30] Meuten T., Hickey A., Franklin K., Grossi B., Tobias J., Newman D.R., Jennings S.H., Correa M., Sannes P.L. (2012). WNT7B in Fibroblastic Foci of Idiopathic Pulmonary Fibrosis. Respir. Res..

[31] Cao H., Chen X., Hou J., Wang C., Xiang Z., Shen Y., Han X. (2020). The Shh/Gli Signaling Cascade Regulates Myofibroblastic Activation of Lung-Resident Mesenchymal Stem Cells via the Modulation of Wnt10a Expression during Pulmonary Fibrogenesis. Lab. Investig..

[32] Lehmann M., Hu Q., Hu Y., Hafner K., Costa R., van den Berg A., Königshoff M. (2020). Chronic WNT/β-Catenin Signaling Induces Cellular Senescence in Lung Epithelial Cells. Cell. Signal..

[33] Noguchi S., Saito A., Nagase T. (2018). YAP/TAZ Signaling as a Molecular Link between Fibrosis and Cancer. Int. J. Mol. Sci..

[34] Samara K.D., Antoniou K.M., Karagiannis K., Margaritopoulos G., Lasithiotaki I., Koutala E., Siafakas N.M. (2012). Expression Profiles of Toll-like Receptors in Non-Small Cell Lung Cancer and Idiopathic Pulmonary Fibrosis. Int. J. Oncol..

[35] Yang H.-Z., Cui B., Liu H.-Z., Chen Z.-R., Yan H.-M., Hua F., Hu Z.-W. (2009). Targeting TLR2 Attenuates Pulmonary Inflammation and Fibrosis by Reversion of Suppressive Immune Microenvironment. J. Immunol..

[36] Larson-Casey J.L., Deshane J.S., Ryan A.J., Thannickal V.J., Carter A.B. (2016). Macrophage Akt1 Kinase-Mediated Mitophagy Modulates Apoptosis Resistance and Pulmonary Fibrosis. Immunity.

[37] Homma S., Nagaoka I., Abe H., Takahashi K., Seyama K., Nukiwa T., Kira S. (1995). Localization of Platelet-Derived Growth Factor and Insulin-like Growth Factor I in the Fibrotic Lung. Am. J. Respir. Crit. Care Med..

[38] Sun Q., Liu L., Mandal J., Molino A., Stolz D., Tamm M., Lu S., Roth M. (2022). PDGF-BB Induces PRMT1 Expression through ERK1/2 Dependent STAT1 Activation and Regulates Remodeling in Primary Human Lung Fibroblasts. Cell. Signal..

[39] Grotendorst G.R. (1997). Connective Tissue Growth Factor: A Mediator of TGF-Beta Action on Fibroblasts. Cytokine Growth Factor Rev..

[40] Selman M., Pardo A. (2021). When Things Go Wrong: Exploring Possible Mechanisms Driving the Progressive Fibrosis Phenotype in Interstitial Lung Diseases. Eur. Respir. J..

[41] Zanin-Silva D.C., Santana-Gonçalves M., Kawashima-Vasconcelos M.Y., Oliveira M.C. (2021). Management of Endothelial Dysfunction in Systemic Sclerosis: Current and Developing Strategies. Front. Med..

[42] Garrett S.M., Hsu E., Thomas J.M., Pilewski J.M., Feghali-Bostwick C. (2019). Insulin-like Growth Factor (IGF)-II-Mediated Fibrosis in Pathogenic Lung Conditions. PLoS ONE.

[43] Klinkhammer B.M., Floege J., Boor P. (2018). PDGF in Organ Fibrosis. Mol. Aspects Med..

[44] Ludwicka A., Ohba T., Trojanowska M., Yamakage A., Strange C., Smith E.A., Leroy E.C., Sutherland S., Silver R.M. (1995). Elevated Levels of Platelet Derived Growth Factor and Transforming Growth Factor-Beta 1 in Bronchoalveolar Lavage Fluid from Patients with Scleroderma. J. Rheumatol..

[45] de Araújo R., Lôbo M., Trindade K., Silva D.F., Pereira N. (2019). Fibroblast Growth Factors: A Controlling Mechanism of Skin Aging. Skin Pharmacol. Physiol..

[46] Spagnolo P., Lee J.S., Sverzellati N., Rossi G., Cottin V. (2018). The Lung in Rheumatoid Arthritis: Focus on Interstitial Lung Disease. Arthritis Rheumatol..

[47] Brito Y., Glassberg M.K., Ascherman D.P. (2017). Rheumatoid Arthritis-Associated Interstitial Lung Disease: Current Concepts. Curr. Rheumatol. Rep..

[48] Yanagihara T., Inoue Y. (2020). Insights into Pathogenesis and Clinical Implications in Myositis-Associated Interstitial Lung Diseases. Curr. Opin. Pulm. Med..

[49] Casciola-Rosen L., Andrade F., Ulanet D., Wong W.B., Rosen A. (1999). Cleavage by Granzyme B Is Strongly Predictive of Autoantigen Status: Implications for Initiation of Autoimmunity. J. Exp. Med..

[50] Grundtman C., Lundberg I.E. (2006). Pathogenesis of Idiopathic Inflammatory Myopathies. Curr. Rheumatol. Rep..

[51] Nguyen X.-X., Nishimoto T., Takihara T., Mlakar L., Bradshaw A.D., Feghali-Bostwick C. (2021). Lysyl Oxidase Directly Contributes to Extracellular Matrix Production and Fibrosis in Systemic Sclerosis. Am. J. Physiol. Lung Cell. Mol. Physiol..

[52] Vadasz Z., Balbir Gurman A., Meroni P., Farge D., Levi Y., Ingegnoli F., Braun-Moscovici Y., Rosner I., Slobodin G., Rozenbaum M. (2019). Lysyl Oxidase-a Possible Role in Systemic Sclerosis-Associated Pulmonary Hypertension: A Multicentre Study. Rheumatology.

[53] Enomoto Y., Suzuki Y., Hozumi H., Mori K., Kono M., Karayama M., Furuhashi K., Fujisawa T., Enomoto N., Nakamura Y. (2017). Clinical Significance of Soluble CD163 in Polymyositis-Related or Dermatomyositis-Related Interstitial Lung Disease. Arthritis Res. Ther..

[54] Horiike Y., Suzuki Y., Fujisawa T., Yasui H., Karayama M., Hozumi H., Furuhashi K., Enomoto N., Nakamura Y., Inui N. (2019). Successful Classification of Macrophage-Mannose Receptor CD206 in Severity of Anti-MDA5 Antibody Positive Dermatomyositis Associated ILD. Rheumatology.

[55] Weigold F., Günther J., Pfeiffenberger M., Cabral-Marques O., Siegert E., Dragun D., Philippe A., Regensburger A.-K., Recke A., Yu X. (2018). Antibodies against Chemokine Receptors CXCR3 and CXCR4 Predict Progressive Deterioration of Lung Function in Patients with Systemic Sclerosis. Arthritis Res. Ther..

[56] Wangkaew S., Euathrongchit J., Wattanawittawas P., Kasitanon N., Louthrenoo W. (2016). Incidence and Predictors of Interstitial Lung Disease (ILD) in Thai Patients with Early Systemic Sclerosis: Inception Cohort Study. Mod. Rheumatol..

[57] Nagy A., Nagy T., Kolonics-Farkas A.M., Eszes N., Vincze K., Barczi E., Tarnoki A.D., Tarnoki D.L., Nagy G., Kiss E. (2021). Autoimmune Progressive Fibrosing Interstitial Lung Disease: Predictors of Fast Decline. Front. Pharmacol..

[58] Watanabe M., Horimasu Y., Iwamoto H., Yamaguchi K., Sakamoto S., Masuda T., Nakashima T., Miyamoto S., Ohshimo S., Fujitaka K. (2019). C-C Motif Chemokine Ligand 15 May Be a Useful Biomarker for Predicting the Prognosis of Patients with Chronic Hypersensitivity Pneumonitis. Respiration.

[59] Prasse A., Probst C., Bargagli E., Zissel G., Toews G.B., Flaherty K.R., Olschewski M., Rottoli P., Müller-Quernheim J. (2009). Serum CC-Chemokine Ligand 18 Concentration Predicts Outcome in Idiopathic Pulmonary Fibrosis. Am. J. Respir. Crit. Care Med..

[60] Tiev K.P., Hua-Huy T., Kettaneh A., Gain M., Duong-Quy S., Tolédano C., Cabane J., Dinh-Xuan A.T. (2011). Serum CC Chemokine Ligand-18 Predicts Lung Disease Worsening in Systemic Sclerosis. Eur. Respir. J..

[61] Khanna D., Denton C.P., Jahreis A., van Laar J.M., Frech T.M., Anderson M.E., Baron M., Chung L., Fierlbeck G., Lakshminarayanan S. (2016). Safety and Efficacy of Subcutaneous Tocilizumab in Adults with Systemic Sclerosis (FaSScinate): A Phase 2, Randomised, Controlled Trial. Lancet.

[62] De Lauretis A., Sestini P., Pantelidis P., Hoyles R., Hansell D.M., Goh N.S.L., Zappala C.J., Visca D., Maher T.M., Denton C.P. (2013). Serum Interleukin 6 Is Predictive of Early Functional Decline and Mortality in Interstitial Lung Disease Associated with Systemic Sclerosis. J. Rheumatol..

[63] Wakamatsu K., Nagata N., Kumazoe H., Oda K., Ishimoto H., Yoshimi M., Takata S., Hamada M., Koreeda Y., Takakura K. (2017). Prognostic Value of Serial Serum KL-6 Measurements in Patients with Idiopathic Pulmonary Fibrosis. Respir. Investig..

[64] Kuwana M., Shirai Y., Takeuchi T. (2016). Elevated Serum Krebs von Den Lungen-6 in Early Disease Predicts Subsequent Deterioration of Pulmonary Function in Patients with Systemic Sclerosis and Interstitial Lung Disease. J. Rheumatol..

[65] Kim H.C., Choi K.H., Jacob J., Song J.W. (2020). Prognostic Role of Blood KL-6 in Rheumatoid Arthritis-Associated Interstitial Lung Disease. PLoS ONE.

[66] Sánchez-Díez S., Munoz X., Ojanguren I., Romero-Mesones C., Espejo D., Villar A., Gómez-Olles S., Cruz M.-J. (2022). YKL-40 and KL-6 Levels in Serum and Sputum of Patients Diagnosed with Hypersensitivity Pneumonitis. J. Allergy Clin. Immunol. Pract..

[67] Ejima M., Okamoto T., Suzuki T., Miyazaki Y. (2022). Role of Serum Surfactant Protein-D as a Prognostic Predictor in Fibrotic Hypersensitivity Pneumonitis. Respir. Investig..

[68] Tzouvelekis A., Herazo-Maya J.D., Slade M., Chu J.-H., Deiuliis G., Ryu C., Li Q., Sakamoto K., Ibarra G., Pan H. (2017). Validation of the Prognostic Value of MMP-7 in Idiopathic Pulmonary Fibrosis. Respirology.

[69] Fraser E., Denney L., Antanaviciute A., Blirando K., Vuppusetty C., Zheng Y., Repapi E., Iotchkova V., Taylor S., Ashley N. (2021). Multi-Modal Characterization of Monocytes in Idiopathic Pulmonary Fibrosis Reveals a Primed Type I Interferon Immune Phenotype. Front. Immunol..

[70] Kreuter M., Lee J.S., Tzouvelekis A., Oldham J.M., Molyneaux P.L., Weycker D., Atwood M., Kirchgaessler K.-U., Maher T.M. (2021). Monocyte Count as a Prognostic Biomarker in Patients with Idiopathic Pulmonary Fibrosis. Am. J. Respir. Crit. Care Med..

[71] Achaiah A., Rathnapala A., Pereira A., Bothwell H., Dwivedi K., Barker R., Iotchkova V., Benamore R., Hoyles R.K., Ho L.-P. (2022). Neutrophil Lymphocyte Ratio as an Indicator for Disease Progression in Idiopathic Pulmonary Fibrosis. BMJ Open Respir. Res..

[72] Naik P.K., Bozyk P.D., Bentley J.K., Popova A.P., Birch C.M., Wilke C.A., Fry C.D., White E.S., Sisson T.H., Tayob N. (2012). Periostin Promotes Fibrosis and Predicts Progression in Patients with Idiopathic Pulmonary Fibrosis. Am. J. Physiol. Lung Cell. Mol. Physiol..

[73] Chen J., Chen Y., Liu D., Lin Y., Zhu L., Song S., Hu Y., Liang T., Liu Y., Liu W. (2022). Predictors of Long-Term Prognosis in Rheumatoid Arthritis-Related Interstitial Lung Disease. Sci. Rep..

[74] Takahashi H., Fujishima T., Koba H., Murakami S., Kurokawa K., Shibuya Y., Shiratori M., Kuroki Y., Abe S. (2000). Serum Surfactant Proteins A and D as Prognostic Factors in Idiopathic Pulmonary Fibrosis and Their Relationship to Disease Extent. Am. J. Respir. Crit. Care Med..

[75] Hamai K., Iwamoto H., Ishikawa N., Horimasu Y., Masuda T., Miyamoto S., Nakashima T., Ohshimo S., Fujitaka K., Hamada H. (2016). Comparative Study of Circulating MMP-7, CCL18, KL-6, SP-A, and SP-D as Disease Markers of Idiopathic Pulmonary Fibrosis. Dis. Markers.

[76] Kohno N., Kyoizumi S., Awaya Y., Fukuhara H., Yamakido M., Akiyama M. (1989). New Serum Indicator of Interstitial Pneumonitis Activity. Sialylated Carbohydrate Antigen KL-6. Chest.

[77] Ohshimo S., Yokoyama A., Hattori N., Ishikawa N., Hirasawa Y., Kohno N. (2005). KL-6, a Human MUC1 Mucin, Promotes Proliferation and Survival of Lung Fibroblasts. Biochem. Biophys. Res. Commun..

[78] Guo L., Yang Y., Liu F., Jiang C., Yang Y., Pu H., Li W., Zhong Z. (2020). Clinical Research on Prognostic Evaluation of Subjects with IPF by Peripheral Blood Biomarkers, Quantitative Imaging Characteristics and Pulmonary Function Parameters. Arch. Bronconeumol..

[79] Zheng P., Zheng X., Takehiro H., Cheng Z.J., Wang J., Xue M., Lin Q., Huang Z., Huang H., Liao C. (2021). The Prognostic Value of Krebs von Den Lungen-6 and Surfactant Protein-A Levels in the Patients with Interstitial Lung Disease. J. Transl. Intern. Med..

[80] Ikeda K., Chiba H., Nishikiori H., Azuma A., Kondoh Y., Ogura T., Taguchi Y., Ebina M., Sakaguchi H., Miyazawa S. (2020). Serum Surfactant Protein D as a Predictive Biomarker for the Efficacy of Pirfenidone in Patients with Idiopathic Pulmonary Fibrosis: A Post-Hoc Analysis of the Phase 3 Trial in Japan. Respir. Res..

[81] Bauer Y., White E.S., de Bernard S., Cornelisse P., Leconte I., Morganti A., Roux S., Nayler O. (2017). MMP-7 Is a Predictive Biomarker of Disease Progression in Patients with Idiopathic Pulmonary Fibrosis. ERJ Open Res..

[82] Ando M., Miyazaki E., Ito T., Hiroshige S., Nureki S., Ueno T., Takenaka R., Fukami T., Kumamoto T. (2010). Significance of Serum Vascular Endothelial Growth Factor Level in Patients with Idiopathic Pulmonary Fibrosis. Lung.

[83] Bonhomme O., André B., Gester F., de Seny D., Moermans C., Struman I., Louis R., Malaise M., Guiot J. (2019). Biomarkers in Systemic Sclerosis-Associated Interstitial Lung Disease: Review of the Literature. Rheumatology.

[84] Tyker A., Ventura I.B., Lee C.T., Strykowski R., Garcia N., Guzy R., Jablonski R., Vij R., Strek M.E., Chung J.H. (2021). High-Titer Rheumatoid Factor Seropositivity Predicts Mediastinal Lymphadenopathy and Mortality in Rheumatoid Arthritis-Related Interstitial Lung Disease. Sci. Rep..

[85] Mouthon L., Bérezné A., Guillevin L., Valeyre D. (2010). Therapeutic Options for Systemic Sclerosis Related Interstitial Lung Diseases. Respir. Med..

[86] Distler O., Assassi S., Cottin V., Cutolo M., Danoff S.K., Denton C.P., Distler J.H.W., Hoffmann-Vold A.-M., Johnson S.R., Müller Ladner U. (2020). Predictors of Progression in Systemic Sclerosis Patients with Interstitial Lung Disease. Eur. Respir. J..

[87] Ashmore P., Tikly M., Wong M., Ickinger C. (2018). Interstitial Lung Disease in South Africans with Systemic Sclerosis. Rheumatol. Int..

[88] Ho K.T., Reveille J.D. (2003). The Clinical Relevance of Autoantibodies in Scleroderma. Arthritis Res. Ther..

[89] Schupp J., Becker M., Günther J., Müller-Quernheim J., Riemekasten G., Prasse A. (2014). Serum CCL18 Is Predictive for Lung Disease Progression and Mortality in Systemic Sclerosis. Eur. Respir. J..

[90] Hoffmann-Vold A.-M., Tennøe A.H., Garen T., Midtvedt Ø., Abraityte A., Aaløkken T.M., Lund M.B., Brunborg C., Aukrust P., Ueland T. (2016). High Level of Chemokine CCL18 Is Associated with Pulmonary Function Deterioration, Lung Fibrosis Progression, and Reduced Survival in Systemic Sclerosis. Chest.

[91] Liu X., Mayes M.D., Pedroza C., Draeger H.T., Gonzalez E.B., Harper B.E., Reveille J.D., Assassi S. (2013). Does C-Reactive Protein Predict the Long-Term Progression of Interstitial Lung Disease and Survival in Patients with Early Systemic Sclerosis?. Arthritis Care Res..

[92] Ross L., Stevens W., Rabusa C., Wilson M., Ferdowsi N., Walker J., Sahhar J., Ngian G.-S., Zochling J., Roddy J. (2018). The Role of Inflammatory Markers in Assessment of Disease Activity in Systemic Sclerosis. Clin. Exp. Rheumatol..

[93] Volkmann E.R., Tashkin D.P., Roth M.D., Clements P.J., Khanna D., Furst D.E., Mayes M., Charles J., Tseng C.-H., Elashoff R.M. (2016). Changes in Plasma CXCL4 Levels Are Associated with Improvements in Lung Function in Patients Receiving Immunosuppressive Therapy for Systemic Sclerosis-Related Interstitial Lung Disease. Arthritis Res. Ther..

[94] Doishita S., Inokuma S., Asashima H., Nakachi S., Matsuo Y., Rokutanda R., Kobayashi S., Hagiwara K., Satoh T., Akiyama O. (2011). Serum KL-6 Level as an Indicator of Active or Inactive Interstitial Pneumonitis Associated with Connective Tissue Diseases. Intern. Med..

[95] Hu Y., Wang L.-S., Jin Y.-P., Du S.-S., Du Y.-K., He X., Weng D., Zhou Y., Li Q.-H., Shen L. (2017). Serum Krebs von Den Lungen-6 Level as a Diagnostic Biomarker for Interstitial Lung Disease in Chinese Patients. Clin. Respir. J..

[96] Sumida H., Asano Y., Tamaki Z., Aozasa N., Taniguchi T., Toyama T., Takahashi T., Ichimura Y., Noda S., Akamata K. (2018). Prediction of Therapeutic Response before and during i.v. Cyclophosphamide Pulse Therapy for Interstitial Lung Disease in Systemic Sclerosis: A Longitudinal Observational Study. J. Dermatol..

[97] Elhai M., Hoffmann-Vold A.M., Avouac J., Pezet S., Cauvet A., Leblond A., Fretheim H., Garen T., Kuwana M., Molberg Ø. (2019). Performance of Candidate Serum Biomarkers for Systemic Sclerosis-Associated Interstitial Lung Disease. Arthritis Rheumatol..

[98] Satoh H., Kurishima K., Ishikawa H., Ohtsuka M. (2006). Increased Levels of KL-6 and Subsequent Mortality in Patients with Interstitial Lung Diseases. J. Intern. Med..

[99] Yanaba K., Hasegawa M., Hamaguchi Y., Fujimoto M., Takehara K., Sato S. (2003). Longitudinal Analysis of Serum KL-6 Levels in Patients with Systemic Sclerosis: Association with the Activity of Pulmonary Fibrosis. Clin. Exp. Rheumatol..

[100] Fukaya S., Oshima H., Kato K., Komatsu Y., Matsumura H., Ishii K., Miyama H., Nagai T., Tanaka I., Mizutani A. (2000). KL-6 as a Novel Marker for Activities of Interstitial Pneumonia in Connective Tissue Diseases. Rheumatol. Int..

[101] Nakanishi Y., Horimasu Y., Yamaguchi K., Sakamoto S., Masuda T., Nakashima T., Miyamoto S., Iwamoto H., Ohshimo S., Fujitaka K. (2021). IL-18 Binding Protein Can Be a Prognostic Biomarker for Idiopathic Pulmonary Fibrosis. PLoS ONE.

[102] Wu C.-Y., Yang H.-Y., Luo S.-F., Lai J.-H. (2021). From Rheumatoid Factor to Anti-Citrullinated Protein Antibodies and Anti-Carbamylated Protein Antibodies for Diagnosis and Prognosis Prediction in Patients with Rheumatoid Arthritis. Int. J. Mol. Sci..

[103] Sato S., Nagaoka T., Hasegawa M., Nishijima C., Takehara K. (2000). Elevated Serum KL-6 Levels in Patients with Systemic Sclerosis: Association with the Severity of Pulmonary Fibrosis. Dermatology.

[104] Nell V.P.K., Machold K.P., Stamm T.A., Eberl G., Heinzl H., Uffmann M., Smolen J.S., Steiner G. (2005). Autoantibody Profiling as Early Diagnostic and Prognostic Tool for Rheumatoid Arthritis. Ann. Rheum. Dis..

[105] Mena-Vázquez N., Godoy-Navarrete F.J., Lisbona-Montañez J.M., Redondo-Rodriguez R., Manrique-Arija S., Rioja J., Mucientes A., Ruiz-Limón P., Garcia-Studer A., Ortiz-Márquez F. (2023). Inflammatory Biomarkers in the Diagnosis and Prognosis of Rheumatoid Arthritis-Associated Interstitial Lung Disease. Int. J. Mol. Sci..

[106] Avouac J., Cauvet A., Steelandt A., Shirai Y., Elhai M., Kuwana M., Distler O., Allanore Y. (2020). Improving Risk-Stratification of Rheumatoid Arthritis Patients for Interstitial Lung Disease. PLoS ONE.

[107] Nakashita T., Ando K., Kaneko N., Takahashi K., Motojima S. (2014). Potential Risk of TNF Inhibitors on the Progression of Interstitial Lung Disease in Patients with Rheumatoid Arthritis. BMJ Open.

[108] Lee Y.S., Kim H.C., Lee B.Y., Lee C.K., Kim M.-Y., Jang S.J., Lee H.S., Moon J., Colby T.V., Kim D.S. (2016). The Value of Biomarkers as Predictors of Outcome in Patients with Rheumatoid Arthritis-Associated Usual Interstitial Pneumonia. Sarcoidosis, Vasc. Diffus. Lung Dis. Off. J. WASOG.

[109] He J., Zhang J., Ren X. (2022). Krebs von Den Lungen-6 as a Clinical Marker for Hypersensitivity Pneumonitis: A Meta-Analysis and Bioinformatics Analysis. Front. Immunol..

[110] Long X., He X., Ohshimo S., Griese M., Sarria R., Guzman J., Costabel U., Bonella F. (2017). Serum YKL-40 as Predictor of Outcome in Hypersensitivity Pneumonitis. Eur. Respir. J..

[111] Johansen J.S., Milman N., Hansen M., Garbarsch C., Price P.A., Graudal N. (2005). Increased Serum YKL-40 in Patients with Pulmonary Sarcoidosis--a Potential Marker of Disease Activity?. Respir. Med..

[112] Vorselaars A.D.M., van Moorsel C.H.M., Zanen P., Ruven H.J.T., Claessen A.M.E., van Velzen-Blad H., Grutters J.C. (2015). ACE and SIL-2R Correlate with Lung Function Improvement in Sarcoidosis during Methotrexate Therapy. Respir. Med..

[113] Cai M., Bonella F., He X., Sixt S.U., Sarria R., Guzman J., Costabel U. (2013). CCL18 in Serum, BAL Fluid and Alveolar Macrophage Culture Supernatant in Interstitial Lung Diseases. Respir. Med..

[114] Bargagli E., Magi B., Olivieri C., Bianchi N., Landi C., Rottoli P. (2011). Analysis of Serum Amyloid A in Sarcoidosis Patients. Respir. Med..

[115] Di Francesco A.M., Verrecchia E., Sicignano L.L., Massaro M.G., Antuzzi D., Covino M., Pasciuto G., Richeldi L., Manna R. (2021). The Use of Chitotriosidase as a Marker of Active Sarcoidosis and in the Diagnosis of Fever of Unknown Origin (FUO). J. Clin. Med..

[116] Kraaijvanger R., Janssen Bonás M., Vorselaars A.D.M., Veltkamp M. (2020). Biomarkers in the Diagnosis and Prognosis of Sarcoidosis: Current Use and Future Prospects. Front. Immunol..

[117] Nombel A., Fabien N., Coutant F. (2021). Dermatomyositis with Anti-MDA5 Antibodies: Bioclinical Features, Pathogenesis and Emerging Therapies. Front. Immunol..

[118] Li X., Liu Y., Cheng L., Huang Y., Yan S., Li H., Zhan H., Li Y. (2022). Roles of Biomarkers in Anti-MDA5-Positive Dermatomyositis, Associated Interstitial Lung Disease, and Rapidly Progressive Interstitial Lung Disease. J. Clin. Lab. Anal..

[119] Porse S., Hoyer N., Shaker S.B. (2022). Impact of Reduction in Antifibrotic Treatment on Mortality in Idiopathic Pulmonary Fibrosis. Respir. Med..

[120] Richeldi L., Kolb M., Jouneau S., Wuyts W.A., Schinzel B., Stowasser S., Quaresma M., Raghu G. (2020). Efficacy and Safety of Nintedanib in Patients with Advanced Idiopathic Pulmonary Fibrosis. BMC Pulm. Med..

[121] Vianello A., Molena B., Turato C., Braccioni F., Arcaro G., Paladini L., Andretta M., Saetta M. (2019). Pirfenidone Improves the Survival of Patients with Idiopathic Pulmonary Fibrosis Hospitalized for Acute Exacerbation. Curr. Med. Res. Opin..

[122] Nathan S.D., Albera C., Bradford W.Z., Costabel U., Glaspole I., Glassberg M.K., Kardatzke D.R., Daigl M., Kirchgaessler K.-U., Lancaster L.H. (2017). Effect of Pirfenidone on Mortality: Pooled Analyses and Meta-Analyses of Clinical Trials in Idiopathic Pulmonary Fibrosis. Lancet Respir. Med..

[123] Fernández Pérez E.R., Crooks J.L., Lynch D.A., Humphries S.M., Koelsch T.L., Swigris J.J., Solomon J.J., Mohning M.P., Groshong S.D., Fier K. (2023). Pirfenidone in Fibrotic Hypersensitivity Pneumonitis: A Double-Blind, Randomised Clinical Trial of Efficacy and Safety. Thorax.

[124] Herrmann F.E., Hesslinger C., Wollin L., Nickolaus P. (2022). BI 1015550 Is a PDE4B Inhibitor and a Clinical Drug Candidate for the Oral Treatment of Idiopathic Pulmonary Fibrosis. Front. Pharmacol..

[125] Richeldi L., Azuma A., Cottin V., Hesslinger C., Stowasser S., Valenzuela C., Wijsenbeek M.S., Zoz D.F., Voss F., Maher T.M. (2022). Trial of a Preferential Phosphodiesterase 4B Inhibitor for Idiopathic Pulmonary Fibrosis. N. Engl. J. Med..

[126] Richeldi L., Azuma A., Cottin V., Kreuter M., Maher T.M., Martinez F.J., Oldham J.M., Valenzuela C., Gordat M., Liu Y. (2023). Design of a Phase III, Double-Blind, Randomised, Placebo-Controlled Trial of BI 1015550 in Patients with Idiopathic Pulmonary Fibrosis (FIBRONEER-IPF). BMJ Open Respir. Res..

[127] Clapp L.H., Gurung R. (2015). The Mechanistic Basis of Prostacyclin and Its Stable Analogues in Pulmonary Arterial Hypertension: Role of Membrane versus Nuclear Receptors. Prostaglandins Other Lipid Mediat..

[128] Nathan S.D., Waxman A., Rajagopal S., Case A., Johri S., DuBrock H., De La Zerda D.J., Sahay S., King C., Melendres-Groves L. (2021). Inhaled Treprostinil and Forced Vital Capacity in Patients with Interstitial Lung Disease and Associated Pulmonary Hypertension: A Post-Hoc Analysis of the INCREASE Study. Lancet Respir. Med..

[129] Nathan S.D., Behr J., Cottin V., Lancaster L., Smith P., Deng C.Q., Pearce N., Bell H., Peterson L., Flaherty K.R. (2022). Study Design and Rationale for the TETON Phase 3, Randomised, Controlled Clinical Trials of Inhaled Treprostinil in the Treatment of Idiopathic Pulmonary Fibrosis. BMJ Open Respir. Res..

[130] Corte T.J., Lancaster L., Swigris J.J., Maher T.M., Goldin J.G., Palmer S.M., Suda T., Ogura T., Minnich A., Zhan X. (2021). Phase 2 Trial Design of BMS-986278, a Lysophosphatidic Acid Receptor 1 (LPA(1)) Antagonist, in Patients with Idiopathic Pulmonary Fibrosis (IPF) or Progressive Fibrotic Interstitial Lung Disease (PF-ILD). BMJ Open Respir. Res..

[131] Maher T.M., Ford P., Brown K.K., Costabel U., Cottin V., Danoff S.K., Groenveld I., Helmer E., Jenkins R.G., Milner J. (2023). Ziritaxestat, a Novel Autotaxin Inhibitor, and Lung Function in Idiopathic Pulmonary Fibrosis: The ISABELA 1 and 2 Randomized Clinical Trials. JAMA.

[132] Zephyrus II: A Phase 3, Randomized, Double-Blind, Placebo-Controlled Efficacy and Safety Study of Pamrevlumab in Subjects with Idiopathic Pulmonary Fibrosis (IPF). https://www.clinicaltrials.gov/study/NCT04419558.

[133] Raghu G., Hamblin M.J., Brown A.W., Golden J.A., Ho L.A., Wijsenbeek M.S., Vasakova M., Pesci A., Antin-Ozerkis D.E., Meyer K.C. (2022). Long-Term Evaluation of the Safety and Efficacy of Recombinant Human Pentraxin-2 (RhPTX-2) in Patients with Idiopathic Pulmonary Fibrosis (IPF): An Open-Label Extension Study. Respir. Res..

[134] Richeldi L., Anstrom K.J., Behr J., Corte T.J., Cottin V., Jenkins G., Kamath N., Inoue Y., Islam L., Nathan S.D. (2021). Recombinant Human Pentraxin-2 for Idiopathic Pulmonary Fibrosis: Design of STARSCAPE-OLE, a Phase III Open Label Extension Study. Eur. Respir. J..

[135] Flaherty K.R., Wells A.U., Cottin V., Devaraj A., Walsh S.L.F., Inoue Y., Richeldi L., Kolb M., Tetzlaff K., Stowasser S. (2019). Nintedanib in Progressive Fibrosing Interstitial Lung Diseases. N. Engl. J. Med..

[136] Distler O., Highland K.B., Gahlemann M., Azuma A., Fischer A., Mayes M.D., Raghu G., Sauter W., Girard M., Alves M. (2019). Nintedanib for Systemic Sclerosis-Associated Interstitial Lung Disease. N. Engl. J. Med..

[137] Solomon J.J., Danoff S.K., Woodhead F.A., Hurwitz S., Maurer R., Glaspole I., Dellaripa P.F., Gooptu B., Vassallo R., Cox P.G. (2023). Safety, Tolerability, and Efficacy of Pirfenidone in Patients with Rheumatoid Arthritis-Associated Interstitial Lung Disease: A Randomised, Double-Blind, Placebo-Controlled, Phase 2 Study. Lancet Respir. Med..

[138] Cottin V., Brown K.K. (2019). Interstitial lung disease associated with systemic sclerosis (SSc-ILD). Respir. Res..

[139] Udwadia Z.F., Mullerpattan J.B., Balakrishnan C., Richeldi L. (2015). Improved Pulmonary Function Following Pirfenidone Treatment in a Patient with Progressive Interstitial Lung Disease Associated with Systemic Sclerosis. Lung India.

[140] Khanna D., Albera C., Fischer A., Khalidi N., Raghu G., Chung L., Chen D., Schiopu E., Tagliaferri M., Seibold J.R. (2016). An Open-Label, Phase II Study of the Safety and Tolerability of Pirfenidone in Patients with Scleroderma-Associated Interstitial Lung Disease: The LOTUSS Trial. J. Rheumatol..

[141] Miura Y., Saito T., Fujita K., Tsunoda Y., Tanaka T., Takoi H., Yatagai Y., Rin S., Sekine A., Hayashihara K. (2014). Clinical Experience with Pirfenidone in Five Patients with Scleroderma-Related Interstitial Lung Disease. Sarcoidosis Vasc. Diffus. Lung Dis. Off. J. WASOG.

[142] Acharya N., Sharma S.K., Mishra D., Dhooria S., Dhir V., Jain S. (2020). Efficacy and Safety of Pirfenidone in Systemic Sclerosis-Related Interstitial Lung Disease-a Randomised Controlled Trial. Rheumatol. Int..

[143] Khanna D., Spino C., Bernstein E., Goldin J., Tashkin D., Roth M. (2022). Combination Therapy of Mycophenolate Mofetil and Pirfenidone vs. Mycophenolate Alone: Results from the Scleroderma Lung Study III. Arthritis & Rheumatology.

[144] Kur-Zalewska J., Kisiel B., Kania-Pudło M., Tłustochowicz M., Chciałowski A., Tłustochowicz W. (2021). A Dose-Dependent Beneficial Effect of Methotrexate on the Risk of Interstitial Lung Disease in Rheumatoid Arthritis Patients. PLoS ONE.

[145] Kim K., Woo A., Park Y., Yong S.H., Lee S.H., Lee S.H., Leem A.Y., Kim S.Y., Chung K.S., Kim E.Y. (2022). Protective Effect of Methotrexate on Lung Function and Mortality in Rheumatoid Arthritis-Related Interstitial Lung Disease: A Retrospective Cohort Study. Ther. Adv. Respir. Dis..

[146] Xu J., Xiao L., Zhu J., Qin Q., Fang Y., Zhang J.-A. (2022). Methotrexate Use Reduces Mortality Risk in Rheumatoid Arthritis: A Systematic Review and Meta-Analysis of Cohort Studies. Semin. Arthritis Rheum..

[147] Baker M.C., Liu Y., Lu R., Lin J., Melehani J., Robinson W.H. (2023). Incidence of Interstitial Lung Disease in Patients with Rheumatoid Arthritis Treated with Biologic and Targeted Synthetic Disease-Modifying Antirheumatic Drugs. JAMA Netw. Open.

[148] Tashkin D.P., Elashoff R., Clements P.J., Goldin J., Roth M.D., Furst D.E., Arriola E., Silver R., Strange C., Bolster M. (2006). Cyclophosphamide versus Placebo in Scleroderma Lung Disease. N. Engl. J. Med..

[149] Tashkin D.P., Roth M.D., Clements P.J., Furst D.E., Khanna D., Kleerup E.C., Goldin J., Arriola E., Volkmann E.R., Kafaja S. (2016). Mycophenolate Mofetil versus Oral Cyclophosphamide in Scleroderma-Related Interstitial Lung Disease (SLS II): A Randomised Controlled, Double-Blind, Parallel Group Trial. Lancet Respir. Med..

[150] Sharma S., Mathew J., Kopp C., Dhir V., Dhooria S., Sinha A., Jain S. (2023). A Randomized Controlled Trial to Compare the Efficacy and Safety of Tacrolimus with Mycophenolate Mofetil in Patients with Systemic Sclerosis—Interstitial Lung Disease (INSIST TRIAL) [Abstract]. Arthritis Rheumatol..

[151] Khanna D., Lin C.J.F., Furst D.E., Goldin J., Kim G., Kuwana M., Allanore Y., Matucci-Cerinic M., Distler O., Shima Y. (2020). Tocilizumab in Systemic Sclerosis: A Randomised, Double-Blind, Placebo-Controlled, Phase 3 Trial. Lancet Respir. Med..

[152] https://rheumatology.org/interstitial-lung-disease-guideline.

[153] Fujisawa T. (2021). Management of Myositis-Associated Interstitial Lung Disease. Medicina.

[154] Baughman R.P., Valeyre D., Korsten P., Mathioudakis A.G., Wuyts W.A., Wells A., Rottoli P., Nunes H., Lower E.E., Judson M.A. (2021). ERS Clinical Practice Guidelines on Treatment of Sarcoidosis. Eur. Respir. J..

[155] Baughman R.P., Gupta R., Judson M.A., Lower E.E., Birring S.S., Stewart J., Reeves R., Wells A.U. (2022). Value of Pulmonary Function Testing Identifying Progressive Pulmonary Disease in Fibrotic Sarcoidosis: Results of a Prospective Feasibility Study. Sarcoidosis Vasc. Diffus. Lung Dis. Off. J. WASOG.

[156] Study Details|Efficacy and Safety of Intravenous Efzofitimod in Patients with Pulmonary Sarcoidosis. https://classic.clinicaltrials.gov/ct2/show/NCT05415137.

[157] Study of Efficacy, Safety and Tolerability of CMK389 in Patients with Chronic Pulmonary Sarcoidosis. https://classic.clinicaltrials.gov/ct2/show/NCT04064242.

[158] ClinicalTrials.gov. A Study to Assess the Efficacy and Safety of Risankizumab in Participants with Ulcerative Colitis. https://clinicaltrials.gov/study/NCT03398135.

[159] Raghu G., Remy-Jardin M., Ryerson C.J., Myers J.L., Kreuter M., Vasakova M., Bargagli E., Chung J.H., Collins B.F., Bendstrup E. (2020). Diagnosis of Hypersensitivity Pneumonitis in Adults. An Official ATS/JRS/ALAT Clinical Practice Guideline. Am. J. Respir. Crit. Care Med..

[160] Morisset J., Johannson K.A., Vittinghoff E., Aravena C., Elicker B.M., Jones K.D., Fell C.D., Manganas H., Dubé B.-P., Wolters P.J. (2017). Use of Mycophenolate Mofetil or Azathioprine for the Management of Chronic Hypersensitivity Pneumonitis. Chest.

[161] Fiddler C.A., Simler N., Thillai M., Parfrey H. (2019). Use of Mycophenolate Mofetil and Azathioprine for the Treatment of Chronic Hypersensitivity Pneumonitis-A Single-Centre Experience. Clin. Respir. J..

[162] Alexandre A.T., Martins N., Raimundo S., Melo N., Catetano Mota P., Bastos H.N.E., Pereira J.M., Cunha R., Guimarães S., Souto Moura C. (2020). Impact of Azathioprine Use in Chronic Hypersensitivity Pneumonitis Patients. Pulm. Pharmacol. Ther..

[163] Adegunsoye A., Oldham J.M., Fernández Pérez E.R., Hamblin M., Patel N., Tener M., Bhanot D., Robinson L., Bullick S., Chen L. (2017). Outcomes of Immunosuppressive Therapy in Chronic Hypersensitivity Pneumonitis. ERJ Open Res..

[164] Ferreira M., Borie R., Crestani B., Rigaud P., Wemeau L., Israel-Biet D., Leroy S., Quétant S., Plantier L., Dalphin J.-C. (2020). Efficacy and Safety of Rituximab in Patients with Chronic Hypersensitivity Pneumonitis (CHP): A Retrospective, Multicentric, Observational Study. Respir. Med..

[165] Behr J., Prasse A., Kreuter M., Johow J., Rabe K.F., Bonella F., Bonnet R., Grohe C., Held M., Wilkens H. (2021). Pirfenidone in Patients with Progressive Fibrotic Interstitial Lung Diseases Other than Idiopathic Pulmonary Fibrosis (RELIEF): A Double-Blind, Randomised, Placebo-Controlled, Phase 2b Trial. Lancet Respir. Med..

